# A novel transgenic mouse model expressing primate-specific nuclear choline acetyltransferase: insights into potential cholinergic vulnerability

**DOI:** 10.1038/s41598-023-30155-4

**Published:** 2023-02-21

**Authors:** H. E. AlQot, R. J. Rylett

**Affiliations:** Department of Physiology and Pharmacology and Robarts Research Institute, Schulich School of Medicine & Dentistry, University of Western Ontario, London, ON N6A 5K8 Canada

**Keywords:** Neuroscience, Physiology, Medical research

## Abstract

The acetylcholine (ACh) synthesizing enzyme choline acetyltransferase (ChAT) is an important cholinergic neuronal marker whose levels and/or activity are reduced in physiological and pathological aging. One isoform of ChAT, 82-kDa ChAT, is expressed only in primates and found primarily in nuclei of cholinergic neurons in younger individuals, but this protein becomes mostly cytoplasmic with increasing age and in Alzheimer’s disease (AD). Previous studies suggest that 82-kDa ChAT may be involved in regulating gene expression during cellular stress. Since it is not expressed in rodents, we developed a transgenic mouse model that expresses human 82-kDa ChAT under the control of an Nkx2.1 driver. Behavioral and biochemical assays were used to phenotype this novel transgenic model and elucidate the impact of 82-kDa ChAT expression. The 82-kDa ChAT transcript and protein were expressed predominantly in basal forebrain neurons and subcellular distribution of the protein recapitulated the age-related pattern found previously in human necropsy brains. Older 82-kDa ChAT-expressing mice presented with better age-related memory and inflammatory profiles. In summary, we established a novel transgenic mouse expressing 82-kDa ChAT that is valuable for studying the role of this primate-specific cholinergic enzyme in pathologies associated with cholinergic neuron vulnerability and dysfunction.

## Introduction

Central cholinergic neurons contribute to regulation of a variety of physiologic functions, such as cognition, attention, locomotion, sleep and immunomodulation^1–5^. In recent years, several studies have focused on the latter function highlighting a cholinergic anti-inflammatory effect on microglia^5–9^. This is dependent on stimulation of nicotinic ACh receptors and is associated with reduced levels of pro-inflammatory cytokines and an overall neuroprotective effect^5,6,10^. Cholinergic neuron dysfunction and neuroinflammation are key hallmarks of aging and correlate with age-dependent cognitive impairment^7,8,11–14^. While no studies connect cholinergic deficits and microglial malfunction in the aged brain directly, several lines of evidence posit a potential interaction. Age-related cholinergic alterations include axonal abnormalities, fibre degeneration, decreased trophic factors and altered expression of ACh receptors, all of which lead to reduced cholinergic signalling^15–17^. This age-dependent cholinergic hypofunction may impair the homeostatic regulation of glial cells causing over activation and chronic inflammation, subsequently exacerbating cholinergic deficits by reducing neuronal trophic factors and leading to synaptic dystrophies and memory impairment^13,18–20^.

A fundamental component of the cholinergic system is the ACh synthesizing enzyme choline acetyltransferase (ChAT) whose levels and activity are reduced in aging and some neurodegenerative diseases^21–24^. In the cholinergic gene locus, ChAT is encoded by a single gene that undergoes alternative splicing and differential utilization of non-coding exons yielding several mRNA polymorphs (R1/2, N1/2, H, S and M) all of which translate to 69-kDa ChAT^25^. In addition, the primate-specific M-transcript has two translation initiation sites leading to the production of both 69- and 82-kDa ChAT proteins in cultured cells, brain and spinal cord^26–28^. The 82-kDa ChAT differs from the 69-kDa ChAT by inclusion of a 118 amino acid extension at the amino-terminus^27^. The relative subcellular distribution of the two protein isoforms is determined by the presence of nuclear localization signals (NLS) and/or nuclear export signals. While 82-kDa and 69-kDa ChAT share a common NLS^29^, 82-kDa ChAT has an additional NLS in its amino-terminal extension imparting preferential nuclear localization^30^. Alternatively, 69-kDa ChAT is considered a nucleocytoplasmic shuttling protein, accumulating primarily in the cytoplasm^30^. Interestingly, this unique nuclear localization of 82-kDa ChAT is altered in aging, mild cognitive impairment (MCI) and AD, shifting to a more diffuse cytoplasmic distribution^28^. These temporal/spatial changes may implicate 82-kDa ChAT in the selective vulnerability of cholinergic neurons that are known to be highly susceptible to the degenerative changes observed in normal and pathological aging, including AD^31^. However, the functional significance of nuclear 82-kDa ChAT and the mechanisms governing the translocation of 82-kDa ChAT between the nucleus and the cytoplasm remain to be explored.

Importantly, studies from our laboratory and others have uncovered evidence for the potential role of 82-kDa ChAT in the regulation of gene expression. Matsuo and colleagues^32^ reported that the nuclear expression of 82-kDa ChAT selectively increased the mRNA and protein levels of the high-affinity choline transporter in neuronal cells. In microarray analysis performed by our laboratory, the expression of 82-kDa ChAT led to altered expression of several genes that encode for amyloid precursor protein (APP) metabolic enzymes and/or their associated regulatory proteins^33^. Additionally, we demonstrated that 82-kDa ChAT may be constitutively associated with chromatin and may contribute to an epigenetic response under cellular stress conditions^34^.

Data reported regarding 82-kDa ChAT indicate a potential role in regulating gene expression and modifying cellular stress responses. Hence, a better understanding of the physiological role of 82-kDa ChAT and the correlation between cellular stress and its subcellular distribution is necessary. Since 82-kDa ChAT is not expressed endogenously in mice, our laboratory generated a transgenic mouse model that expresses human 82-kDa ChAT to facilitate study of this protein in vivo. The objective of this study is to characterize these mice and establish a behavioural and biochemical profile for this transgenic model.

## Methods and materials

### Mice, genotyping and experimental design

Study design, methods, and experimental procedures were approved by the Committee for the Care and Use of Laboratory Animals at the University of Western Ontario that aligns with the Canadian Council on Animal Care regulations and conform to the ARRIVE guidelines.

**Figure 1:**
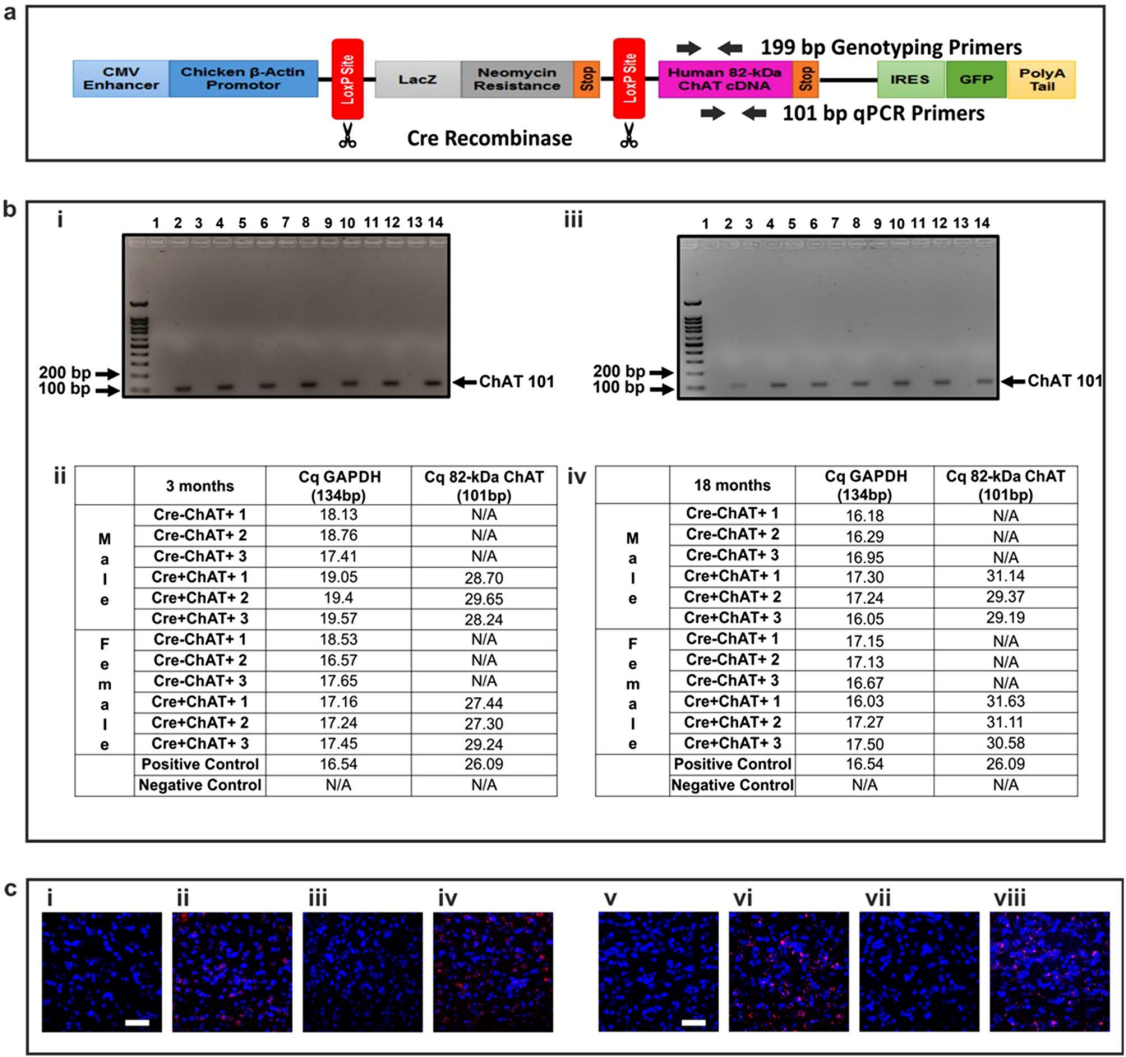
Plasmid encoding the human 82-kDa ChAT transgene and detection of the human M-ChAT transcript encoding 82-kDa ChAT in transgenic mice. (**a**) A diagram of the floxed piZeg vector encoding the human 82-kDa ChAT cDNA in which the second translation initiation site is mutated to preclude formation of 69-kDa ChAT protein. Mice carrying this plasmid only encode 82-kDa ChAT transgene without expressing it due to the floxed LacZ sequence and stop codon between the promotor and the 82-kDa ChAT sequence. In the presence of Cre recombinase, the floxed LacZ sequence is excised, allowing for expression of the 82-kDa ChAT transcript and protein. Both the genotyping and qPCR primers amplify product within the cDNA sequence of the 82-kDa ChAT. (**b**) Representative gels and tables showing the absence or presence of the expected 101 bp amplicon in the basal forebrain of young (i,ii) and old mice (iii,iv), respectively. Lanes 1, 3, 5, 7, 9 and 11 are samples from Cre-ChAT+, lanes 2, 4, 6, 8 10 and 12 are samples from Cre+ChAT+ mice, lane 13 is a negative control and lane 14 is positive control. The positive control used was obtained from human SH-SY5Y cells stably expressing 82-kDa ChAT. (**c**) Confocal images of RNAscope experiments with *in-situ* localization of a probe binding to human 82-kDa ChAT transcripts in the medial septal nuclei. Red dots signify detection of human M-ChAT transcripts in Cre+ChAT+ (ii: young male, iv: young female, vi: old male, viii: old female) mice, but not Cre-ChAT+ (i: young male, iii: young female, v: old male, vii: old female) mice. Mice were either 3 or 18 months of age. Scale bars represent 100 µm. Representative images are from n = 3 mice per sex per genotype.

Transgenic mice were developed at London Regional Gene Targeting and Transgenic Facility on a C57BL/6 background using a plasmid with the cDNA encoding human 82-kDa ChAT that was ligated to a floxed piZeg vector (pcCALL2:82-ChAT, Fig. 1a). Heterozygous mice are crossed with Nkx2.1 Cre-mice (Tg(Nkx2-1-cre)2Sand/C57BL/6 J-Tg, Jackson Laboratories, #008661) to drive expression of the transgene in forebrain neurons. Mice were housed in groups of three in conditions compliant with Canadian Council on Animal Care guidelines (22–25 °C, 50% humidity, 12 h light/dark cycle) with free access to chow and water, and were provided with enrichments (nestlets, cardboard shavings and amber houses). For genotyping, genomic DNA was extracted from ear notches using RED-Extract-N-Amp Tissue PCR kit (Sigma-Aldrich) and amplified using primers for Cre (forward: *AGCCGAAATTGCCAGGATCA*, reverse: *AACCAGCGTTTTCGTTCTGC*) and the 82-kDa ChAT transgene (forward: *TGTCTGAGTACTGGCTGAATGAC*, reverse: *TTGGCACAGTCAGTGGGAATG*). Amplicons were separated on 2% agarose gels and positive bands at 99 bp and 199 bp indicated Cre-positive and ChAT-positive samples, respectively. In this study, 168 mice were allocated by sex, genotype and age in experiments, with 12–14 mice used in longitudinal behavioral studies, 4 mice for histological analysis, 4 mice for RNA analysis and 4 mice for protein analysis. Experimental endpoints were set at 3 and 18 months of age, at which time mice were euthanized and brains recovered for subsequent analysis. For qPCR, arrays and immunoblot experiments, mice were euthanized by cervical dislocation and brains were dissected quickly on ice into cerebral cortex, hippocampus, striatum and basal forebrain, then snap frozen in liquid nitrogen and stored at − 80 °C for subsequent testing. Alternatively, for microscopy studies (protein by immunohistochemistry and mRNA by RNAscope), mice were anaesthetized by intraperitoneal (IP) injection of thiopental (100 mg/kg)/xylazine (5 mg/kg), then perfused trans-cardially with ice-cold phosphate-buffered saline (PBS) for five min followed by ice-cold 4% paraformaldehyde (PFA) in PBS for 10 min. The brains were collected and post-fixed in 4% PFA in PBS at 4 °C for 24 h. Subsequently, brains were transferred to 30% sucrose in PBS at 4 °C for 48–72 h for cryoprotection. The brains were then embedded in Tissue-Plus™ O.C.T. Compound (Thermo, #23–730-571), frozen using dry ice, and stored at − 80 °C until sectioning. Frozen brains were sectioned using a Leica CM350 cryostat into 40 μm free-floating coronal sections and collected in PBS. Sections were transferred into an antigen preserving solution (1% polyvinylpyrrolidone (PVP), 50% ethylene glycol, PBS) and stored at − 20 °C until further processing.

### Neurobehavioral studies

A battery of behavior tests was performed to phenotype mice that were found by genotyping to be Cre-negative and ChAT-positive (called Cre-ChAT+) and Cre-positive and ChAT-positive (called Cre+ChAT+). At age 2.5-months, 24–28 mice (12–14 males and females each) were entered into behavioral testing, with tests performed between 9 am and 12 pm over a 5-week period in the following order: open field test, grip force strength, Barnes maze and finally Morris water maze, starting at age 3-months. Mice were re-tested at age 18-months. The experimenter was blinded to the genotype of the test groups. Data compiled from males and females was analyzed separately to determine if there were possible sex-related effects.

### Habituation and general activity

Habituation and exploratory activity were assessed using the open field arena test. Mice were placed in locomotor chambers (20 × 20 × 30 cm), covered with a perforated lid and activity was recorded by a network of infra-red beams and analyzed by VersaMax 2.0 Fusion software (Accuscan Instruments, Inc., Columbus, OH, USA). Lights were dimmed for the duration of the test. Activity was monitored for 2 h, divided into twelve 10-min intervals, on each of 3 consecutive days. The parameters measured included total distance, time spent at margin and time spent at centre. For habituation, parameters from the twelve 10-min intervals were summed for each day and compared over the 3-day test period using two-way RM ANOVA-Tukey. To assess general and exploratory activity, only parameters acquired during the first 10-min observation period of the first day were analyzed using one-way ANOVA-Sidak.

### Weight and motor function

Following the open field arena test, mice underwent a grip force strength test using a wire bar and a digital force gauge. Briefly, mice were allowed to grip the wire bar using their forelimbs and then the maximum force (N) required for release was determined. Each mouse performed 5 trials with 5-min inter-trial intervals. Mice were weighed and, to assess grip force strength (N/kg), the average force value (N) from 5 trials was normalized to the mouse’s weight (kg). Statistical significance was determined using one-way ANOVA-Sidak.

### Learning and memory

#### Barnes maze

The maze consisted of a table with a 92-cm diameter white flat circular platform raised to 105-cm above ground with 20 equally spaced potential target escape holes around the perimeter. Four visual clues were placed at equal distances around the maze and a buzzer was used as the aversive stimulus to motivate escape into the target escape box. At the beginning of each trial, mice were placed in an opaque cylindrical chamber for 10-s. On the first training session, mice underwent an adaptation trial where they were trained to explore the maze and guided to the position of the target escape box where they were kept for 2-min. Following this, mice were trained over 4 trials (3-min/trial) each day for 4 consecutive days with mice allowed to remain for 1-min in the target escape box after finding it. Mice that failed to find the target escape hole at the end of the 3-min period were guided to it and kept there for 1-min. Each mouse had an inter-trial interval of at least 15-min and the maze was cleaned with 70% ethanol between mice to avoid odor clues. For each test, mice were divided into 2 counterbalanced groups and each group assigned a different target escape hole. Target escape hole locations assigned when the mice were aged 3 months were different from those assigned when the mice were aged 18 months to avoid interferences from previous acquisitions. Parameters recorded during the training trials included primary errors (errors prior to visiting target escape hole), primary latency (time to first visit to target) and escape latency (time to first escape/entrance into the target escape box). These were analyzed using two-way RM ANOVA-Tukey. At 24 h after the last training trial (day 5), a probe trial was conducted with the target escape hole closed and mice were allowed to explore the maze for 90 s. This trial was repeated on day 12. Probe trial measures included the preference index (PI, calculated as the number of target visits divided by the average number of non-target visits) and the time spent at the target escape hole versus the average time spent at non target escape holes with significance determined using one-way ANOVA-Sidak or two-way ANOVA-Tukey, respectively. All training and probe trials were videotaped and analyzed using Anymaze video tracking software (Stoelting).

### Morris water maze

The paradigm consisted of a circular tank 120-cm diameter and 50-cm deep filled with water to a depth of 40-cm at a temperature of 20 ± 2 °C. The maze was divided virtually into 4 quadrants (northeast, northwest, southeast and southwest) by a north–south and east–west axis with a 10-cm diameter escape platform submerged 1.5-cm below the water in the centre of the target quadrant. Four visual clues, distinct from those used in the Barnes maze, were placed at equal distances around the water tank. During acquisition, the mice were trained to locate the escape platform over 4 trials (90 s/trial) each day for 4 consecutive days while probe trials included a short-term (day 5) and a long-term (day 12) probe trial. When the mouse found the escape platform, it was allowed to remain there for 30-s. Mice that failed to find the platform at the end of the 90-s were guided to it and kept there for 30-s. After each swim, mice were allowed to dry in individual boxes where they were kept for a minimum of 15-min until subsequent trials. The starting location for each trial on any given day was randomized and like the Barnes maze, mice were divided into 2 groups and each group assigned an opposite target quadrant to offset potential quadrant effects. Target locations assigned when mice were aged 3 months were different from those assigned when mice were aged 18 months to avoid interferences from previous acquisitions. Parameters recorded during the training trials included distance, speed and escape latency (time to reach platform) with these analyzed using two-way RM ANOVA-Tukey. On day 5 and day 12 probe trials, the platform was removed and mice were allowed to explore the water maze starting at the location opposite to where the platform was previously. Time spent in each quadrant along with path efficiencies were recorded and statistical significance was calculated using two-way ANOVA-Sidak. All training and probe trials were videotaped and analyzed using Anymaze video tracking software (Stoelting).

### Quantitative real time PCR and PCR arrays

For assessment of 82-kDa ChAT transcript, total RNA was extracted from basal forebrain using Aurum Total RNA Fatty and Fibrous Tissue Kit (BioRad). Subsequently, cDNA was prepared using iScript gDNA Clear cDNA Synthesis Kit according to the manufacturer’s instructions. Quantitative PCR was performed on a BioRad CFX Connect System using SsoAdvanced Universal SYBR Green Supermix (BioRad) and primers specific for the ChAT M-transcript (forward: *CAACGAGGACGAGCGTTTG*, reverse: *GGTTGGTGGAGTCTTTCACGAG,* amplicon size:101 bp). For each group and genotype, four male and four female mice were used. Samples were run in triplicate and average Ct was used to calculate ΔCt and subsequently ΔΔCt for fold change analysis. GAPDH was used as a reference gene and statistical analysis was performed based on ΔCt values.

PCR arrays probing aging pathways (84 genes, 5 reference genes and 7 controls) were performed on 3- and 18-month old mice with an n = 3 mice/sex/genotype/age. Cerebral cortex tissues were homogenized using a hand-held homogenizer and total RNA extracted using the RNeasy Plus Mini Kit (Qiagen, 74134). To synthesize cDNA, the RT2 First Strand Kit (Qiagen, 330404) and RNase-Free DNase kit (Qiagen, 79254) were used to eliminate genomic DNA contamination. PCR reactions were performed using RT2 SYBR Green qPCR master mix (Qiagen, 330503) and RT2 Profiler PCR Array Kit for Aging (PAMM-178Z) according to manufacturer’s instructions on a BioRad CFX Connect System. Analysis of gene expression data was performed using Qiagen’s online platform (RT2 Profiler PCR Arrays & Assays Data Analysis software (https://geneglobe.qiagen.com/ca/analyze). All genes were normalized to a minimum of three reference genes. Data showing at least a twofold change with a p-value ≤ 0.05 were considered significant.

### Immunoblots

Tissues were dispersed by sonication in N-PER Neuronal Protein Extraction Reagent (Thermo, 87792) at a ratio 1:7.5, supplemented with protease and phosphatase inhibitors (Roche). Lysates were incubated at 4 °C for 1 h, then insoluble debris was sedimented by centrifugation at 14,000 rpm for 15 min at 4 °C. Supernatants were transferred to fresh tubes and protein concentration measured using Pierce BCA Protein assay kit (Thermo, 23225) with bovine serum albumin as standard. Proteins were denatured by heating at 95 °C for 10 min in Laemmli buffer (2% SDS, 10% glycerol, 0.1 M sodium phosphate buffer, pH 7.2, 0.001% bromophenol blue and 2.5% β-mercaptoethanol). Equal amounts of protein/sample (25 μg) were separated on 8% or 12% SDS-PAGE gels and transferred to polyvinylidene fluoride (PVDF) membranes using wet transfer method. Membranes were blocked in 5% non-fat dry milk or 5% normal goat serum in Tris-buffered saline (TBS) with 0.1% Tween-20 (0.1% TBST) for 1 h at room temperature, then incubated overnight at 4 °C with primary antibodies (Table 1) diluted in blocking buffer. The next day, blots were washed using 0.1% TBST and incubated with secondary antibodies against mouse, rabbit or rat (Table 1) diluted in blocking buffer (1:5000–1:10,000) for 1 h at room temperature. BioRad Clarity Western ECL substrate was used to detect immunoreactive bands that were imaged using a Chemidoc imaging system. Densitometric analysis was performed on immunoreactive bands using Image Lab software (BioRad) and normalized to the loading control β-actin.

**Table 1:** Primary and secondary antibodies used for immunoblots and immunofluorescence.

Antibody	Vendor/Catalogue no	Dilution for immunoblots	Dilution for immunohistochemistry
Custom rabbit Anti-82 kDa ChAT (C-terminus)^29^	GeneMed synthesis	–	1:100
Custom rabbit Anti-82 kDa ChAT (N-terminus)^35^	GeneMed synthesis	–	1:100
Rabbit Anti-Iba1	Abcam ab178846	1:1000	1:1000
Mouse Anti-NeuN (clone A60) AlexaFluor 555 conjugate	Millipore-Sigma MAB377A5	–	1:1000
Rabbit Anti-iNOS	Thermo scientific PA1-036	1:1000	–
Mouse Anti-Cluster differentiation 86 (CD86)	Thermo scientific MA515697	1:1000	–
Rabbit Anti-Cluster differentiation 206 (CD206)	Abcam ab125028	1:1000	–
Rabbit Anti-Arg1	Proteintech 16001-1-AP	1:1000	–
β-Actin	Millipore-Sigma a5441	1:1000	–
Rabbit Anti-complement C1q	Thermo scientific PA5-29586	1:1000	–
Goat Anti-complement C3	Thermo scientific PA129715	1:1000	–
Rabbit Anti-IL1 β	Abcam ab2105	1:1000	–
Rabbit Anti-IL6	Abcam ab208113	1:1000	–
Rabbit Anti-IL4	Thermo scientific PA5-25165	1:1000	–
Goat anti-rabbit IgG (H + L) cross-adsorbed secondary antibody, Alexa Fluor 488	Invitrogen A11008	–	1:500/1:1000
Goat anti-rabbit IgG (H + L) highly cross-adsorbed Alexa Fluor 568	Invitrogen A11036	–	1:500/1:1000
HRP-conjugated goat Anti-Rabbit IgG (H + L)	BioRad 170-6515	1:5000/1:10000	–
HRP-conjugated goat Anti-Mouse IgG (H + L)	Jackson ImmunoResearch 115-035-003	1:5000/1:10000	–
HRP-conjugated donkey Anti-Goat IgG (H + L)	Jackson ImmunoResearch 705-035-003	1:5000/1:10,000	

### Immunohistochemistry and imaging

Immunofluorescence analysis of proteins of interest were conducted on free-floating coronal sections (40 μm) obtained from perfusion fixed brains. Briefly, sections were washed with 1X tris-buffered saline (TBS) for 1 h, then permeabilized in 100% methanol at − 20 °C for 10 min and washed with 1X TBS. Sections were blocked in 4% normal donkey serum (Abcam, ab7475) in 1X TBS with 0.05% Triton X-100 for 1.5–2 h at room temperature. Next, sections were incubated with primary antibodies (Table 1) diluted in 2% normal donkey serum in 1X TBS with 0.05% Triton, overnight with shaking at room temperature. Sections were then washed with 1X TBS and incubated with corresponding secondary antibodies (Table 1) in 2% normal donkey serum in 1X TBS with 0.05% Triton for 2 h at room temperature. Lastly, sections were washed with 1X TBS, mounted on Superfrost plus-charged slides (Thermo Scientific # 12-550-15), air dried and cover-slipped with Vectashield hardset mounting medium with DAPI (Vectashield, VECTH1500). Tiled whole brain scans were acquired using a Thermo-Evos imaging system at 20X (dry) magnification. Higher resolution tiled and individual images of the medial septum, cortex and cerebellum were obtained on a Leica TSC-SP8 confocal microscope using 20X (dry), 40X (oil immersion) or 63X (oil immersion) objectives. Z-stacking for higher magnification fields was set at a step-size of 1–4 µm. Leica Applications Suite (LAS X) and Image J software were used for image processing and analysis. Image analysis was based on 3 sections per mouse with a minimum acquisition of 3 individual fields. Morphological analysis of microglia, including branch length and count, were performed using the skeleton analysis plug-in in Image J, as described by Young and Morrison^36^.

### RNAscope in-situ hybridization

Frozen-fixed brain sections (40 µm) were mounted on Superfrost plus-charged slides, air-dried for 2 h and probed using RNAscope Multiplex Fluorescent Reagent Kit (Advanced Cell Diagnostics #323133), according to the manufacturer’s instructions. Briefly, brain sections on slides were washed in PBS, fixed in 10% neutral buffered formalin solution (Millipore-Sigma #HT5011-1CS) at 4 °C for 15 min, dehydrated, then pretreated with hydrogen peroxide at room temperature for 10 min and washed with Milli-Q water. Target retrieval was performed by boiling in protease III at 98-102 °C for 5 min, followed by Milli-Q water wash and dehydration. Pretreated slides were then hybridized with probes specific to 82-kDa ChAT mRNA (Hs-CHAT-No-XMm-O1-C1, 1:1000) for 2 h at 40 °C in a RNAscope HybEZ oven. This was followed by three sequential amplifications and HRP channel development and fluorophore application (Opal 690, 1:15,000) at 40 °C in a RNAscope HybEZ oven. Finally, slides were counterstained with DAPI, air dried, cover-slipped using ProLong Gold Antifade mounting reagent (Invitrogen # P36934) and allowed to dry overnight in the dark at room temperature. Positive and negative mouse-specific RNAscope 3-plex controls were used in parallel with test probes to validate the assay. Stacked images (4 µm/step) were acquired on a Leica TSC-SP8 confocal microscope using a 40X (oil immersion) objective for high resolution images of the medial septal nuclei. Whole brain sections were imaged using a Thermo-Evos imaging system at 20X (dry) magnification. Two sections from 2 mice/sex/genotype/age were imaged with a minimum acquisition of 3 fields and processing was performed on a Leica Applications Suite (LAS X) and Image J software.

### Statistical analysis

Data analysis was performed using GraphPad Prism software version 9 and is presented as mean ± standard deviation (SD). Densitometric and immunofluorescence quantifications were analysed using one-way ANOVA with Sidak post-hoc test. Measurements were compared between genotypes, sexes and across different age points. Statistical significance was set at P ≤ 0.05.

## Results

### Mouse breeding, genotyping and classification

The 82-kDa ChAT isoform is a primate-specific protein that is not expressed endogenously in mice, thus to study function of the cholinergic neuronal protein our laboratory developed a transgenic mouse model with neuron-specific expression in the forebrain using the Cre-Lox recombination system^37^. Transgenic mice were developed on a C57BL/6 background using a piZEG vector^38^ to encode the human 82-kDa ChAT cDNA (pcCALL2:82-ChAT) (Fig. 1a). These mice carry the 82-kDa ChAT transgene, but do not express it due to the floxed stop codon preceding the transgene and can therefore be used as transgenic control mice. Founder mice carrying the 82-kDa ChAT transgene were identified by genotyping using primers targeted specifically to the human ChAT cDNA; these primers do not bind to genomic ChAT in mice. Subsequently, carrier mice were crossed with Nkx2.1 Cre-mice (C57BL/6 J-Tg, Jackson Laboratories) that are designed to express Cre-recombinase in telencephalic neurons that express the Nkx2.1 transcription factor^39^. In mice expressing the Cre and 82-kDa ChAT transcripts, expression of the Cre recombinase protein leads to excision of the floxed stop codon, enabling transcription of the ChAT transgene giving production of mice with neuron-specific expression in the forebrain region. Based on the presence or absence of the 82-kDa ChAT transgene and/or Cre recombinase, mice were allocated into two groups, controls that carry the 82-kDa ChAT cDNA without the Cre transgene called *Cre-ChAT+ *and 82-kDa ChAT mice that carry both 82-kDa ChAT and Cre transgenes called *Cre*+*ChAT+ *.

### Investigating the presence of 82-kDa ChAT transcript

Nkx2.1 is a transcription factor expressed in cholinergic neurons and some other neurons in cortex, hippocampus and subcortical telencephalon^39^. As many cholinergic neuron cell bodies are localized to the basal forebrain, 82-kDa ChAT mRNA and protein were evaluated in this region. Expression of the primate-specific ChAT mRNA was assessed in the basal forebrain by qPCR and RNAscope using primers and probes, respectively, designed to specifically detect the human, but not mouse, ChAT transcript. qPCR of basal forebrain samples from Cre+ChAT+ mice showed significant amplification of a 101 bp product indicating the presence of the 82-kDa ChAT mRNA. This amplicon was not detected in tissue obtained from the Cre-ChAT+ mice (Fig. 1b i-iv). When comparing transcript expression levels in young and old Cre+ChAT+ mice, there was a significant decrease in the fold-change of 82-kDa ChAT in 18-month old mice compared to 3-month old mice (males: − 0.88 ± 0.10, p ≤ 0.05, females: − 0.83 ± 0.10, p ≤ 0.05 (Supp. Fig. 1a). To further confirm this finding and to assess expression of the transcript for the 82-kDa ChAT transgene using an alternative experimental approach, we used RNAscope with a probe customized to detect and visualize the human M-ChAT transcript that encodes 82-kDa ChAT in coronal sections obtained from transgenic mice. Positive fluorescent signals were observed in the medial septum of Cre+ChAT+ (Fig. 1c ii, iv, vi, viii), but not in Cre-ChAT+ mice (Fig. 1c i, iii, v, vii), confirming the presence and absence of the 82-kDa ChAT transcript in these mice, respectively. To confirm that the transgene expression was restricted to brain areas containing cholinergic neuronal perikarya, RNAscope in situ hybridization was performed on sections of cerebellum from Cre+ChAT+ mice with this revealing an absence of fluorescent signals in this brain region (Supp. Fig. 1b).

### Investigating the expression pattern of 82-kDa ChAT protein and its subcellular localization

**Figure 2:**
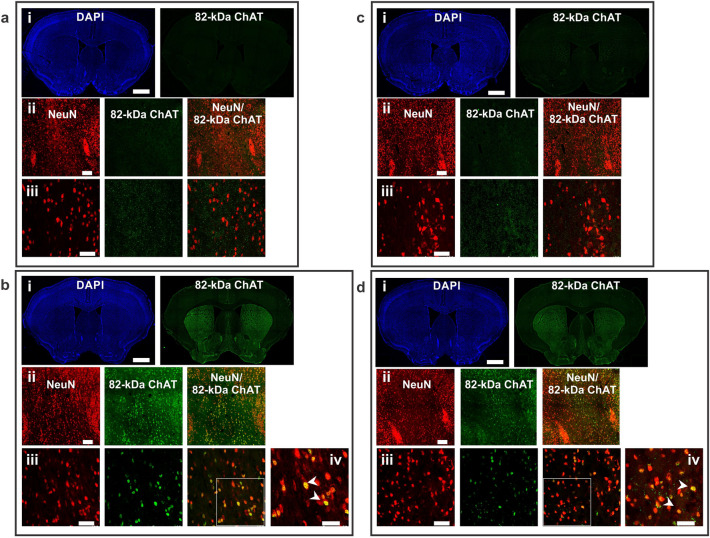
The regional and subcellular distribution pattern of 82-kDa ChAT in 3-month old Cre-ChAT+ and Cre+ChAT+ mice. Coronal sections (40 μm) of whole brain show 82-kDa ChAT distribution in the basal forebrain of Cre-ChAT+ male ((**a**)-i), Cre+ChAT+ male ((**b**)-i), Cre-ChAT+ female ((**c**)-i), and Cre+ChAT+ female ((**d**)-i) mice. Representative confocal images of 82-kDa ChAT immunoreactivity in the medial septum of Cre-ChAT+ (males: (**a**)-ii and iii, females: (**c**)-ii and iii) and Cre+ChAT+ (males: (**b**)-ii and iii and females: (**d**)-ii and iii) mice. Immunopositive signals detected in Cre+ChAT+ sections ((**b**) and (**d**), ii-iv) appear to be primarily nuclear as indicated by the yellow colour seen in the merged panels (white arrowheads in (**b**)-iv and (**d**)-iv) due to co-localization of 82-kDa ChAT (green) with the neuronal nuclei marker NeuN (red). White square insets represent the enlarged images (1.5X) in panels (**b**)-iv and (**d**)-iv. All images were obtained using either 10X, 20X or 40X objectives (n = 3/sex/genotype/age). Scale bars for the panels are as follows; 250 µm for i and ii, 100 µm for iii and 50 µm for iv.

**Figure 3:**
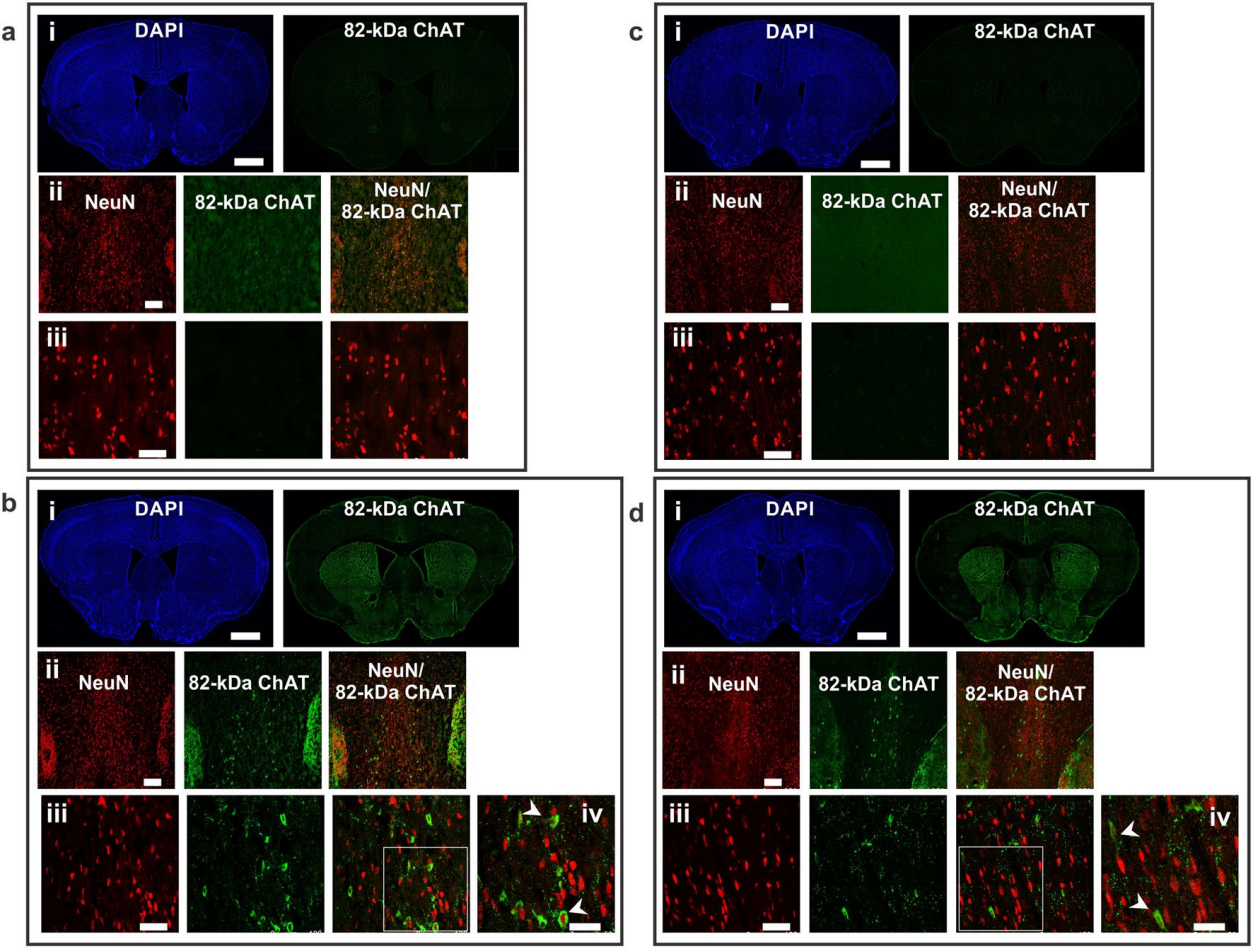
The regional and subcellular distribution pattern of 82-kDa ChAT in 18-month old Cre-ChAT+ and Cre+ChAT+ mice. Coronal sections (40 μm) of whole brain show 82-kDa ChAT distribution in the basal forebrain of Cre-ChAT+ male ((**a**)-i), Cre+ChAT+ male ((**b**)-i), Cre-ChAT+ female ((**c**)-i), and Cre+ChAT+ female ((**d**)-i) mice. Representative confocal images of 82-kDa ChAT immunoreactivity in the medial septum of Cre-ChAT+ (males: (**a**)-ii and iii, females: (**c**)-ii and iii) and Cre+ChAT+ (males: (**b**)-ii and iii and females: (**d**)-ii and iii) mice. Immunopositive signals detected in Cre+ChAT+ sections ((**b**) and (**d**), ii-iv) appear to be more diffuse within the cell with higher intensity in the cytoplasm of medial septum neurons. This is indicated by the reduced yellow signal in the merged panels, with distinct green fluorescence surrounding red-stained nuclei (white arrowhead in b-iv and d-iv). White square insets represent the enlarged images (1.5X) in panels b-iv and d-iv. All images were obtained using either 10X, 20X or 40X objectives (n = 3/sex/genotype/age). Scale bars for the panels are as follows; 250 µm for i and ii, 100 µm for iii and 50 µm for iv.

The expression and distribution of 82-kDa ChAT protein in brain was investigated by immunofluorescence using custom antibodies that recognize either the C-terminus (CTab) (Figs. 2 and 3) or N-terminus of human ChAT (NTab) (Supp. Fig. 2a,b), but do not recognize mouse ChAT^35^. Immunopositive cells were observed mainly in the medial septum and diagonal band of Broca and to a lesser extent in the lateral septum, cortex and striatum of Cre+ChAT+ mice (Figs. 2b-i and d-i, 3b-i and d-i), but not Cre-ChAT+ mice (Figs. 2a-i and c-i, 3a-i and c-i). Human ChAT immunopositive cells were observed in medial septum of both young (3 month) (Fig. 2b-ii and d-ii) and old (18 month) (Fig. 3b-ii and d-ii) Cre+ChAT+ mice. We also examined subcellular distribution of the 82-kDa ChAT protein within neurons in the medial septum. Importantly, we found that in young mice, staining for the transgenic protein is found predominantly in neuronal nuclei as indicated by co-localization of the human specific antibody (CTab) with NeuN, the neuronal nuclei marker (Fig. 2b and d, iii,iv). By comparison in older mice, 82-kDa ChAT fluorescence appears to be more diffuse in the cytoplasm with minimal staining in the nucleus (Fig. 3b and d, iii,iv). No differences were observed in the localization and subcellular distribution of the protein between male (Figs. 2b, 3b) and female mice (Figs. 2d, 3d). As a negative control, brain sections from cerebellum, which is not known to express Nkx2.1^40^, of young Cre+ChAT+ mice were stained with the 82-kDa ChAT specific antibody and immunopositive cells were not observed (Supp. Fig. 2c).

### Neurobehavioral studies in Cre+ChAT+ mice at 3 and 18 months of age

The neurobehavioral performance of 82-kDa ChAT expressing mice was assessed using a battery of tests to look at general behaviour and locomotion (open field arena), physical attributes (grip force, weight) and aspects of learning and memory (Barnes maze and Morris water maze) at both 3 and 18 months of age.

In the open field arena, mice were observed over the course of 2 h on each of three consecutive days and general activity was assessed by analyzing the first 10 min interval of the first day. At age 3 months, mice habituated normally to the environment over the three days as expected, moving and exploring less as indicated by the decrease in distance moved and time spent at margin and in the centre, accordingly (Supp. Fig. 3). Due to familiarity at the subsequent test point at age 18 months, none of the measured parameters changed over the test period as the arena was no longer considered a new environment. No significant differences were observed between the genotypes or sexes in habituation or general activity. Within genotypes, age differences were noted when analyzing the initial 10 min interval, where 18-month old mice showed a significant decrease in total distance travelled in both sexes and genotypes compared to 3 month old mice.

In the grip force test, no significant genotype differences were observed in forelimb strength, force and weight across the ages tested (Supp. Fig. 4). Only age and sex differences within genotype were noted in forelimb strength and weight, but not force. Younger mice showed significantly higher grip strength and lower weight relative to older mice. Additionally, male mice at both ages showed lower grip strength and higher body weight compared to female mice.

Hippocampal-dependent spatial learning and memory was probed using the Barnes maze, a dry land maze, and the Morris water maze, a wet land maze. Across genotypes, ages and sexes no significant differences were observed in either of the mazes (Supp. Figs. 5, 6). All mice learned the tasks and were able to navigate the mazes to locate the targets effectively as indicated by the decrease in errors, distance and escape latencies over the 4 days of training. Memory performances were similar between all mice tested at the different time points, with mice showing higher preference to target (Barnes maze) and spending more time at the target quadrant relative to other quadrants (Morris water maze) in both short- and long-term probe trials. In the older mice, subtle, but significant, differences were observed between genotypes in retention. Importantly, memory decay, measured as the difference in time spent at target/preference index between probes on days 5 and 12, was significantly higher in male and female Cre-ChAT+ mice relative to Cre+ChAT+ mice (Supp. Fig. 7).

### Aging PCR array in young and old mice

**Figure 4:**
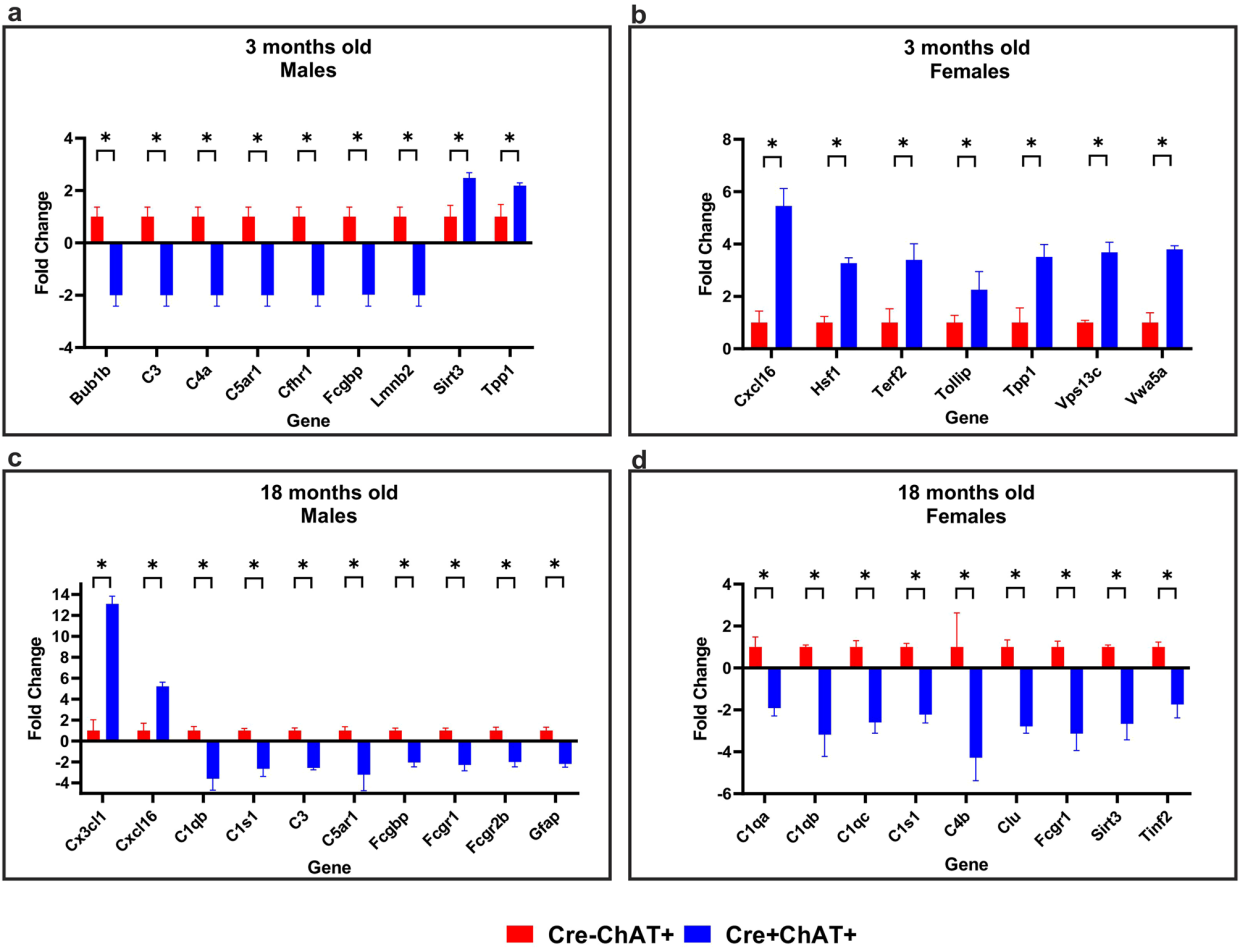
Impact of expression of 82-kDa ChAT on aging-related genes was assessed by qPCR array of RNA from brains of 3- and 18-month old Cre+ChAT+ and age-matched control mice. Cortical RNA was probed for 84 genes related to aging pathways with differences in expression of at least twofold reported. Panels: (**a**) 3-month old male mice (2/84 genes increase, 7/84 genes decrease), (**b**) 3-month old female mice (7/84 genes increase), (**c**) 18-month old male mice (2/84 genes increase, 8/84 genes decrease) and (**d**) 18-month old female mice (9/84 genes decrease). Fold-change analysis was performed using Geneglobe’s online platform (RT^2^ Profiler PCR Data Analysis). Data shown as fold-change ± SD was obtained from 3 independent experiments for each sex and genotype. Statistical analysis was performed using 2-way ANOVA-Sidak and asterisk denote a p-value ≤ 0.05 relative to control. Red bars represent control Cre-ChAT+ mice and blue bars represent Cre+ChAT+ mice.

We performed a qPCR array (RT^2^ Profiler PCR Array Mouse Aging, Qiagen/Geneglobe) on cortical tissues from Cre-ChAT+ and Cre+ChAT+ mice that probes changes in gene expression related to molecular pathways that are known to be implicated in the hallmarks of physiological aging^41^. The genes tested by the array fall broadly under the following categories: nucleic acid stability, binding and repair, epigenetic and transcriptional alterations, telomere attrition, mitochondrial dysfunction, oxidative stress, proteostasis, inflammatory response, neurodegeneration and synaptic transmission. Using Geneglobe’s RT^2^ Profiler PCR Data Analysis platform to determine changes above twofold change with a p-value ≤ 0.05, we found that in the younger age group approximately 10% of the genes probed had either increased or decreased expression in 82-kDa ChAT expressing mice compared to control mice. Similarly, at 18 months of age about 11% of the genes tested showed altered expression levels relative to the control mice. At 3 months of age, 82-kDa ChAT expressing mice showed significant decreases in expression of genes related to inflammation, specifically complement cascade components (C3, C4, C5ar1). Alternatively, genes involved in proteostasis (Hsf1, Vps13c) and telomere attrition (Tpp1, Terf2) had elevated expression in young Cre+ChAT+ mice. Older Cre+ChAT+ cohorts showed significant decreases in expression of inflammatory genes (C1q, C3, C4, C5ar1) and increases in expression of genes implicated in neuron-glia communication and neuroprotection (CxCl16, Cx3Cl1) (Fig. 4).

### Investigating microglia morphology

**Figure 5:**
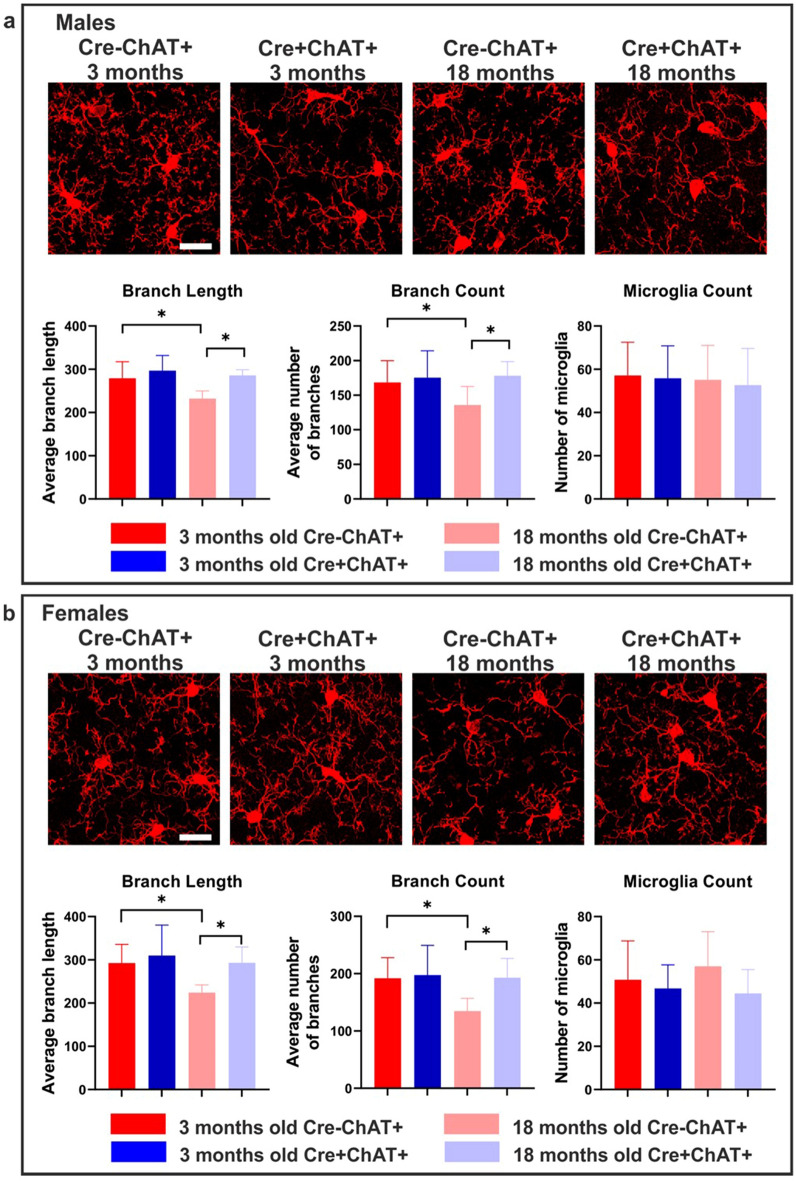
Morphology and branching of microglia in the cortex of young and old Cre-ChAT+ and Cre+ChAT+ mice. High-power magnification images of Iba1 immunofluorescence of 3- and 18-month old mice show age-dependent morphological alterations. Microglia in young Cre-ChAT+ mice (males: (**a**), females: (**b**)) and Cre+ChAT+ mice (males: (**a**), females: (**b**)) exhibit long and slender ramifications. In older Cre-ChAT+ mice (males: (**a**), females: (**b**)), microglia show fragmented branches with significantly shorter length and reduced numbers compared to younger Cre-ChAT+ mice and age-matched Cre+ChAT+ mice. By comparison, 18-month old Cre+ChAT+ mice maintain morphology and branching similar to young Cre+ChAT+ mice as indicated by the branch lengths and count. Image acquisition was obtained with a 63X objective with Z-stacking (stack size: 1 µm, number of stacks is 40–42) of 6 independent fields per mouse. Data were collected from 3 independent experiments/sex/genotype and is represented as mean ± SD. Statistical analysis was performed using one-way ANOVA-Sidak and asterisk denote p-value ≤ 0.05 relative to control. Red bars represent control mice, blue bars represent 82-kDa ChAT-expressing mice, dark shades are 3-month old mice and light shades are 18-month old mice. Scale bars represent 30 µm.

Given the changes observed in expression of inflammation-related genes in the PCR array, we investigated whether there are genotype-related differences in microglia. First, we examined morphology using the microglial marker ionized calcium binding adaptor molecule 1 (Iba1) in the cortex and hippocampus (Fig. 5). At 3 months of age, microglial count, number of branches and branch length were similar in Cre-ChAT+ (Fig. 5a,b) and Cre+ChAT+ mice (Fig. 5a,b). By comparison, 18-month old Cre-ChAT+ mice (Fig. 5a,b) showed significantly lower branching and shorter branch length relative to age-matched 82-kDa ChAT expressing mice (Fig. 5a,b). Moreover, control mice, but not the 82-kDa ChAT expressing mice, showed significant age-dependent de-ramification as indicated by the lower number and length of branches in older mice compared to younger mice (Fig. 5a,b). No significant differences were observed in microglial count at any of the ages tested or between the control and Cre+ChAT+ mice (Fig. 5a,b).

**Figure 6:**
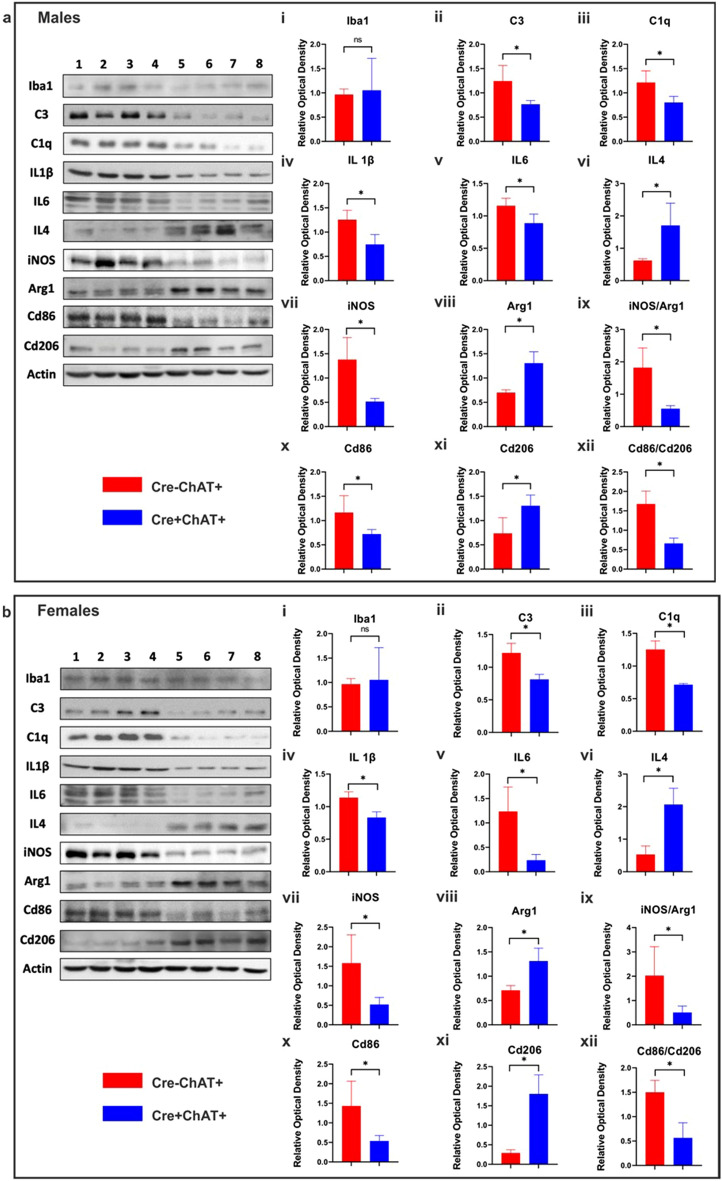
Pro-inflammatory and anti-inflammatory markers were assessed by immunoblot in 18-month old Cre+ChAT+ and control mice. Cortical lysates (25 µg) from male (**a**) and female (**b**) mice were separated using 8 or 12% SDS-PAGE gels, then transferred to PVDF membranes and probed with antibodies against target proteins. Lanes 1–4 represent Cre-ChAT+ mice and lanes 5–8 represent Cre+ChAT+ mice. The 82-kDa ChAT expressing mice exhibit higher levels of markers associated with neuroprotection and anti-inflammation and lower levels of pro-inflammatory markers. Representative blots show both male (**a**) and female (**b**) Cre+ChAT+ mice exhibit lower levels of C3 (ii), C1q (iii), Il1 beta (iv), Il6 (v), iNOS (vii) and Cd86 (x) and higher levels of Il4 (vi), Arg1 (viii) and Cd206 (xi) compared to control mice, without significant differences in the level of the microglial marker, Iba1 (i) between the two genotypes. Additionally, ratios of iNOS/Arg1 (ix) and Cd86/Cd206 (xii) are significantly lower in Cre+ChAT+ mice. Immunopositive bands from 4 biological replicates/sex/genotype were analyzed by densitometry and all samples were normalized to the loading control beta-actin. Data are shown as mean ± SD and statistical analysis was performed using unpaired Student’s *t*-test with asterisks denoting *p ≤ 0.05 relative to control. Original immunoblots for (**a**) male and (**b**) female mice are presented in Supplementary Figs. 8 and 9, respectively.

Next, we analyzed the inflammatory status using immunoblots to assess possible changes in the microglial marker Iba1, complement factors (C1q and C3), pro-inflammatory markers (iNOS, Cd86, IL6, IL1β) and anti-inflammatory markers (Arg1, Cd206, IL4). While no significant differences were noted in the level of Iba1 between Cre-ChAT+ and Cre+ChAT+ mice (Fig. 6a,b, i), several of the other markers showed significant changes between the two genotypes. Consistent with data from the PCR array, 18-month old Cre-ChAT+ mice exhibited higher levels of the complement cascade proteins C3 and C1q compared to age matched Cre+ChAT+ mice (Fig. 6a,b, ii,iii). Additionally, older control mice had significant increases in levels of IL1β (Fig. 6a,b, iv), IL6 (Fig. 6a,b, v), iNOS (Fig. 6a,b, vii) and Cd86 (Fig. 6a,b, x) along with lower levels of IL4 (Fig. 6a,b, vi), Arg1 (Fig. 6a,b, viii) and Cd206 (Fig. 6a,b, xi) compared to Cre+ChAT+ mice. While some of the markers showed only slight or no change, upon analyzing the ratio of these markers (iNOS/Arg1 and Cd86/Cd206), control mice demonstrated significantly higher ratios relative to 82-kDa ChAT expressing mice (Fig. 6a,b, ix and xii, respectively).

## Discussion

We have successfully created a transgenic mouse model with neuronal expression of the primate-specific cholinergic neuronal protein 82-kDa ChAT that recapitulates the regional distribution and subcellular localization of this protein observed in humans^28^ by using an adaptation of the Cre-lox recombination system^37^ and Nkx2.1-Cre driver mice^39^.

The presence of the human M-ChAT transcript encoding 82-kDa ChAT protein in basal forebrain neurons of Cre+ChAT+ mice was demonstrated using qPCR and RNAscope technology. Immunohistochemical analysis confirmed protein expression predominantly in cholinergic neuronal cell bodies of the basal forebrain and striatum of Cre+ChAT+ mice, but not in Cre-ChAT+ mice. Additionally, mRNA and protein expression were not detected in Cre+ChAT+ mice outside the telencephalon, for example in the cerebellum. Our results are consistent with the expression of Nkx2.1^39^ and coincide with the reported distribution of cholinergic neurons in the mammalian brain^42^.

Neurobehavioral profiling revealed no differences between the two genotypes of mice in general activity, motor strength and hippocampal-dependent cognition. In the current study, 82-kDa ChAT expressing and non-expressing mice showed typical age-dependent physiological changes and neurobehavioral alterations, including increased weight gain, decreased general activity and reduced overall strength reported for rodents previously^43,44^. While deficits in hippocampal-dependent memory in aged mice is documented in the literature^45^, it was not observed in the current studies. This could be attributed to the longitudinal experimental design as past studies demonstrated that repeated training and previous test-experience may lead to improved performance in aspects of learning and memory consequently masking age-related deificts^46^. Aging is also associated with accelerated memory decay^47^ which was evident in 18-month old control mice, but interestingly not observed in Cre+ChAT+ mice. While further investigation is required to understand the role of 82-kDa ChAT expression in these cognitive changes, some of this may be attributed to the enhanced expression of CXCL16, a chemokine reported to promote neuronal survival^48^ and modulate presynaptic release of neurotransmitters in the hippocampus^49^. Moreover, the preserved integrity of microglial branching in the aged 82-kDa ChAT expressing mice may also contribute to this behavioral outcome given the role of microglia in synaptic plasticity, neuronal circuitry and consequently cognitive functions^50^.

We investigated the subcellular distribution of 82-kDa ChAT in the basal forebrain of Cre+ChAT+ mice and detected an immunopositive signal localized predominantly to neuronal nuclei of some neurons. In rodents and primates, the immunoreactivity of the canonical smaller isoform of ChAT (69-kDa) is located primarily in cytoplasm and is observed in the soma and projections of cholinergic neurons^51^. By comparison, the larger primate-specific 82-kDa ChAT protein is found mainly in neuronal cell bodies within the nucleus^28,29^. The subcellular distribution of ChAT is determined by the presence of NLS that enable trafficking across the nuclear pore complex^29,52^. The 69-kDa and 82-kDa isoforms of ChAT share a common NLS found at residues 358–368 and 481–486, respectively, allowing for their translocation into the nucleus^29^. However, 69-kDa ChAT is a nucleocytoplasmic shuttling protein that is capable of translocating back across the nuclear envelope via a nuclear export sequence-dependent export pathway (leptomycin B-sensitive Crm-1 pathway), thereby accumulating primarily in the cytoplasm. Alternatively, 82-kDa ChAT is preferentially localized to the nucleus because of a second NLS located within the first nine amino acids of its amino terminus^30^. This unique cellular compartmentalization has been demonstrated in non-neuronal cell lines^29^, neuronal cell lines^30,32^, as well as human necropsy brain and spinal cord tissues^28^. Among the proposed mechanisms that may govern this differential distribution of ChAT is phosphorylation which is known to regulate the nuclear shuttling of proteins^53^. Several protein kinases have been found to phosphorylate 69-kDa and 82-kDa ChAT^35^ and it has been found that a peripheral isoform of ChAT (49-kDa pChAT) has increased nuclear translocation upon inhibition of protein kinase C (PKC)^54^. Interestingly, we found altered expression in genes related to some isoforms of PKC in the 82-kDa ChAT expressing transgenic mouse model (unpublished data).

When comparing immunoreactivity for 82-kDa ChAT in younger and older Cre+ChAT+ mice, we found that in the former, the protein was predominantly nuclear while in the latter it assumed a more cytoplasmic distribution. This recapitulates our findings using human necropsy brains where in young subjects 82-kDa ChAT was localized to the nucleus of neurons, while in older cognitively intact individuals and Alzheimer’s disease patients, it was found in the nucleus and cytoplasm of neurons^28^. The mechanism leading to this age-dependent change in subcellular localization is unknown, but several reports have shown that both normal and pathological aging can be associated with aberrant partitioning of proteins between the nuclear and cytoplasmic compartments. In some cases, this appears to be due to decreased stability and increased permeability of the nuclear pore complex leading to perturbations in the nuclear import/export dynamics^55^. Importantly, aging studies have documented both increased export^56^ and reduced import^57^ of nuclear proteins leading to abnormal cytoplasmic accumulation and subsequent interference with normal cellular functions.

The function of 82-kDa ChAT remains unknown, but we and others have uncovered evidence for its potential role in regulation of gene expression under normal^32^ and cellular stress conditions^33,34^. In the present study, a PCR array focused on pathways related to the aging process revealed altered transcription levels of genes related to protein homeostasis and inflammation in young and old Cre+ChAT+ mice compared to their age-matched control mice, respectively. Similarly, microarray analysis^33^ and ChIP-seq^34^ data from our laboratory using cells expressing 82-kDa ChAT showed altered transcription of genes related to cellular stress response and transcription regulation. Matsuo and colleagues^32^ reported that the overexpression of 82-kDa ChAT in SH-SY5Y neuronal cells selectively increased mRNA and protein levels of the high affinity choline uptake transporter CHT1. Whether 82-kDa ChAT affects gene expression directly by serving as a transcription factor and binding to DNA through its putative DNA binding sites^34,58^ or indirectly by modifying transcription factors or nuclear histones by altering acetylation states^59^ or nuclear choline reserves^60^ remains to be explored. A previous study from our laboratory demonstrated that 82-kDa ChAT may associate constitutively with chromatin thereby stimulating an epigenetic response by altering accessibility of chromatin loops to transcription factors and activators/repressors^34^.

Given the changes observed in inflammation-related genes, we investigated microglial morphology and inflammatory profiles in Cre-ChAT+ and Cre+ChAT+ mice. Aging is associated with chronic inflammation and aged microglia acquire a pro-inflammatory phenotype with reduced arborization, motility and exacerbated response to injury which can impact the homeostatic functions of microglia^61^. Interestingly, older 82-kDa ChAT expressing mice exhibited higher levels of anti-inflammatory markers and lacked the typical age-related de-ramification and heightened pro-inflammatory status seen in age-matched control mice.

In our model, 82-kDa ChAT is expressed in neurons with no known expression in microglia, suggesting that the microglial changes observed in this mouse model may be the result of neuron-glia cross communication. Neuron-glia interactions are facilitated by chemokines (CXCL12, CXCL16, CX3CL1 and CCL2)^62^ as well as complement proteins (C1q and C3)^63^ many of which are expressed in cholinergic neurons and are implicated in their crosstalk with microglia^64,65^. Among the genes showing altered expression in the 82-kDa ChAT mouse were genes related to chemotaxis (CXCL16) and complement cascade (C1q and C3). Recent studies have shown that high C3 levels are associated with a pro-inflammatory phenotype while inhibiting the C3 axis was associated with an anti-inflammatory/neuroprotective profile^66,67^. In the current study, reduced levels of complement proteins in Cre+ChAT+ mice are accompanied by increased expression of anti-inflammatory (IL4, Cd206, Arg1) and reduced expression of pro-inflammatory (iNOS, Cd86, IL1β, IL60)^68^ markers. This, combined with the morphological changes observed in microglia in the Cre+ChAT+ mouse, suggests that 82-kDa ChAT may be involved in mediating communication between cholinergic neurons and microglia.

Several studies have documented the bidirectional relationship between cholinergic neurons and microglia^13^. Neuroinflammation is associated with downregulation of nicotinic ACh receptors, and cholinergic stimulation leads to reduced production of pro-inflammatory mediators and microglial activation^13^. As such, it has been postulated that loss of cholinergic function with increasing age could impact microglial activation leading to the dysfunctional phenotype observed with advanced age^13,65^. With regard to the current mouse model, changes in expression of multiple genes related to the expression of 82-kDa ChAT may have promoted maintenance of the function of cholinergic neurons and impacted communication with microglia. This could possibly impact the age-induced inflammatory milieu by promoting the homeostatic functions of microglia leading to reduced glial dystrophies. This requires further investigation.

### Conclusion and significance

In summary, we established a transgenic mouse model with basal forebrain neuron expression of primate-specific 82-kDa ChAT that shows age-related changes in expression and subcellular localization of the protein similar to that observed in humans. The present study supports previous evidence for a role of 82-kDa ChAT in modifying gene expression and sheds light on a potential role as a modulator of neuron-glia interactions in neuroinflammation. Further studies are required to confirm this directly and to investigate its possible involvement in age-related microglial function. This transgenic mouse model will also be a useful tool to study the physiological function of the primate-specific 82-kDa ChAT and its role in cholinergic neuron vulnerability in physiological aging and neurodegenerative diseases.

## Supplementary Information

Supplementary Figures.

## Data Availability

The datasets generated and analysed during the current study are available from the corresponding author on reasonable request.

## Abbreviations

ACh: Acetylcholine
AD: Alzheimer’s disease
APP: Amyloid precursor protein
ChAT: Choline acetyltransferase
Iba1: Ionized calcium binding adaptor molecule 1
MCI: Mild cognitive impairment
PKC: Protein kinase C
NLS: Nuclear localization signal
