# Supplementary material for: A novel transgenic mouse model expressing primate-specific nuclear choline acetyltransferase: insights into potential cholinergic vulnerability
Source: Sci Rep. 2023 Feb 21;13:3037. doi: 10.1038/s41598-023-30155-4 (PMC9944276; doi:10.1038/s41598-023-30155-4)
Supplement: Supplementary file 1 — Supplementary Figures. [file 41598_2023_30155_MOESM1_ESM.pdf]

Supplementary Figures and Legends

Supplementary Figure 1. (a) Quantification of the fold-change of the 82-kDa ChAT transcript measured by qPCR using RNA prepared from basal forebrain of older mice relative to younger mice. Data were obtained from 4 biological replicates for each sex and age group. Graphs show mean ± SD with asterisks denoting $p \leq 0.05$. Statistical analysis was performed using two-way ANOVA-Sidak. (b) RNAscope *in-situ* hybridization of the human 82-kDa ChAT probe in the cerebellum. Absence of positive signals indicate the lack of 82-kDa ChAT expression in this anatomical region consistent with the reported expression pattern of the Nkx2.1 promotor. Data from RNAscope experiments were collected from 2 independent replicates of males and females. Scale bars in (i) represent 250 μm and in (ii) 100 μm.

Supplementary Figure 2. (a-b) Representative micrographs showing 82-kDa ChAT immunopositive results using the human ChAT N-terminus antibody (NTab) in young (a) and old (b) mice. Cre-ChAT+ mice do not show NTab-positive results (males: a-i and b-i, females: a-iii and b-iii), whereas Cre+ChAT+ mice show NTab-positive results in young (males: a-ii, females: a-iv) and old mice (males: b-ii, females: b-iv) with patterns similar to those seen with CTab in Figures 2 and 3, respectively. Data were collected from 2-3 mice/sex/genotype/age. Scale bars represent 100 μm. (c) Confocal images of coronal brain sections (40 μm) of the cerebellum of 3-month old Cre-ChAT+ (i) and Cre+ChAT+ (ii) mice showing the lack of staining with the CTab antibody (green). This is in agreement with previous reports indicating that the expression of the Nkx2.1 is limited to the telencephalon[39-40]. Scale bars represent 250 μm.

Supplementary Figure 3. Graphs demonstrate habituation of mice in the open field arena reflected by the total distance travelled, time spent at the margins and time spent at the center, recorded for two h over three consecutive days. Both Cre-ChAT+ and Cre+ChAT+ mice appear to habituate normally over the period of three days, moving less and spending more time at the margins rather than the center of the arena. Data are shown for males (a, i-iii) and females (b, i-iii) for 82-kDa ChAT expressing mice and littermate controls at ages 3 and 18 months (n=12-14/sex/genotype/age, results expressed as mean ± SD, * $p \leq 0.05$ day effect, # $p \leq 0.05$ age effect, 2-way RM ANOVA, Tukey post-hoc test). General activity was also assessed over a 10 min interval on the first day (males: a, iv-vi, females: b, iv-vi). No genotype differences were observed and both control and 82-kDa ChAT expressing mice showed age-dependent decreases in activity (males: a-iv, females: b-iv). Results are expressed as mean ± SD for n=12-14/sex/genotype/age, * $p \leq 0.05$ two-way ANOVA-Sidak.

Supplementary Figure 4. Graphs represent the forelimb grip strength (i), force (ii) and body weight (iii) of male (a) and female (b) 82-kDa ChAT expressing mice and littermate control mice at ages 3 and 18 months, averaged over five consecutive trials. Grip strength normalized to body weight [Force (N)/Weight (Kg)] was calculated using the force (N) and weight (Kg) parameters. Only age-dependent effects were observed, including reduced strength and increased weight in older mice compared to younger mice. Results are expressed as mean ± SD for n=12-14/sex/genotype/age, * $p \leq 0.05$, one-way ANOVA-Sidak.

Supplementary Figure 5. Learning and memory performance in the Barnes maze. Graphs showing the primary errors (i) and escape latency (ii) recorded over four days of training (four

trials/day) for male (a) and female (b) 82-kDa ChAT expressing mice and littermate control mice at ages 3 and 18 months. For both ages, both genotypes learned the task effectively and were able to locate the escape box as indicated by the decrease in error frequency and latency over the course of the training days. Results are expressed as mean ± SD for n=12-14/sex/genotype/age, * $p \leq 0.05$ day-effect, 2-way RM ANOVA, Tukey post-hoc test. (iii-vi). With regard to memory performance, graphs show the preference index (target visits/average non-target visits, iii and v) and target visits (iv and vi) during the short-term probe trial (probe 1, day 5, iii-iv) and long-term probe trial (probe 2, day 12, v-vi) for male (a) and female (b) 82-kDa ChAT expressing mice and littermate control mice at ages 3 and 18 months. No genotype effects were observed in 3-month old mice. At 18 months of age, male and female 82-kDa ChAT expressing mice showed significantly higher target visits compared to control mice in the second (a-vi) and first probe trial (b-iv), respectively. Results are expressed as mean ± SD for n=12-14/sex/genotype/age, * $p \leq 0.05$ genotype effect, 2-way ANOVA-Sidak.

Supplementary Figure 6. Learning and memory performance in Morris water maze. Graphs showing the escape latency (i), distance travelled (ii), and speed (iii) recorded during the acquisition phase over four days (4 trials/day) for male (a) and female (b) 82-kDa ChAT expressing mice and littermate control mice at ages 3 and 18 months. No significant differences were observed in the learning curves between Cre-ChAT+ and Cre+ ChAT+ or between 3- and 18-month old mice. The two genotypes at both ages were able to learn the location of the hidden platform as indicated by the decrease in latency and distance travelled over the four days. Swim speed remained unchanged over the course of training at both ages, with the older mice swimming at a relatively slower speed compared to younger mice. Results are expressed as mean ± SD for n=12-

14/sex/genotype/age, *p $\leq$ 0.05 day-effect, 2-way RM ANOVA, Tukey post-hoc test. Memory performance was assessed using time spent in each quadrant (T= Target, O= Opposite, L= Left and R= Right) during the short-term probe trial (probe 1, day 5, iv) and long-term probe trial (probe 2, day 12, v) for male (a) and female (b) 82-kDa ChAT expressing mice and littermate controls at ages 3 and 18 months. In both probe trials, mice show significant retention of the quadrant in which the escape platform was hidden with no differences between genotypes or ages. Results are expressed as mean ± SD for n=12-14/sex/genotype/age, *p $\leq$ 0.05 quadrant effect, 2-way ANOVA, Tukey post-hoc test.

Supplementary Figure 7. Decay of memory retention. Recall of the target location, measured as time spent at the target, was compared between the probe trials at day 5 (short-term recall) and day 12 (long-term recall) in mice at ages 3 (i-ii, v-vi) and 18 months (iii-iv, vii-viii) in the Barnes maze (BM) and Morris water maze (MWM). Young Cre-ChAT+ (i and v) and Cre+ChAT+ (ii and vi) maintained the same level of retention between the probe trials in the BM (i-iv) and MWM (v-viii). Older Cre-ChAT+ mice (iii and vii) showed significant decay in memory over time as indicated by the shorter time spent at target on day 12 compared to day 5. On the other hand, older Cre+ChAT+ mice (iv and viii) maintained similar retention with no significant decline in memory retention between the 2 probe trials. Results are expressed as mean ± SD for n=12-14/sex/genotype/age, * p $\leq$ 0.05 probe effect, unpaired t-test.

Supplementary Figure 8. Original immunoblots for pro- and anti-inflammatory markers in male mice shown in the cropped images included in Figure 6a. Uncropped blots probing for Iba1, C3,

C1q, IL1β, IL6, IL4, iNOS, Arg1, Cd86 and Cd206 (left panels) and their corresponding loading control β-actin (right panels) for cortical lysates from male Cre-ChAT+ (lanes 2-5) and Cre+ChAT+ mice (lanes 6-9). The red boxes indicate the cropped regions shown in Figure 6a.

Supplementary Figure 9. Original immunoblots for pro- and anti-inflammatory markers in female mice shown in the cropped images included in Figure 6b. Uncropped blots probing for Iba1, C3, C1q, IL1β, IL6, IL4, iNOS, Arg1, Cd86 and Cd206 (left panels) and their corresponding loading control β-actin (right panels) for cortical lysates from female Cre-ChAT+ (lanes 2-5) and Cre+ChAT+ mice (lanes 6-9). The red boxes indicate the cropped regions shown in Figure 6b.

Supplementary Figure 1

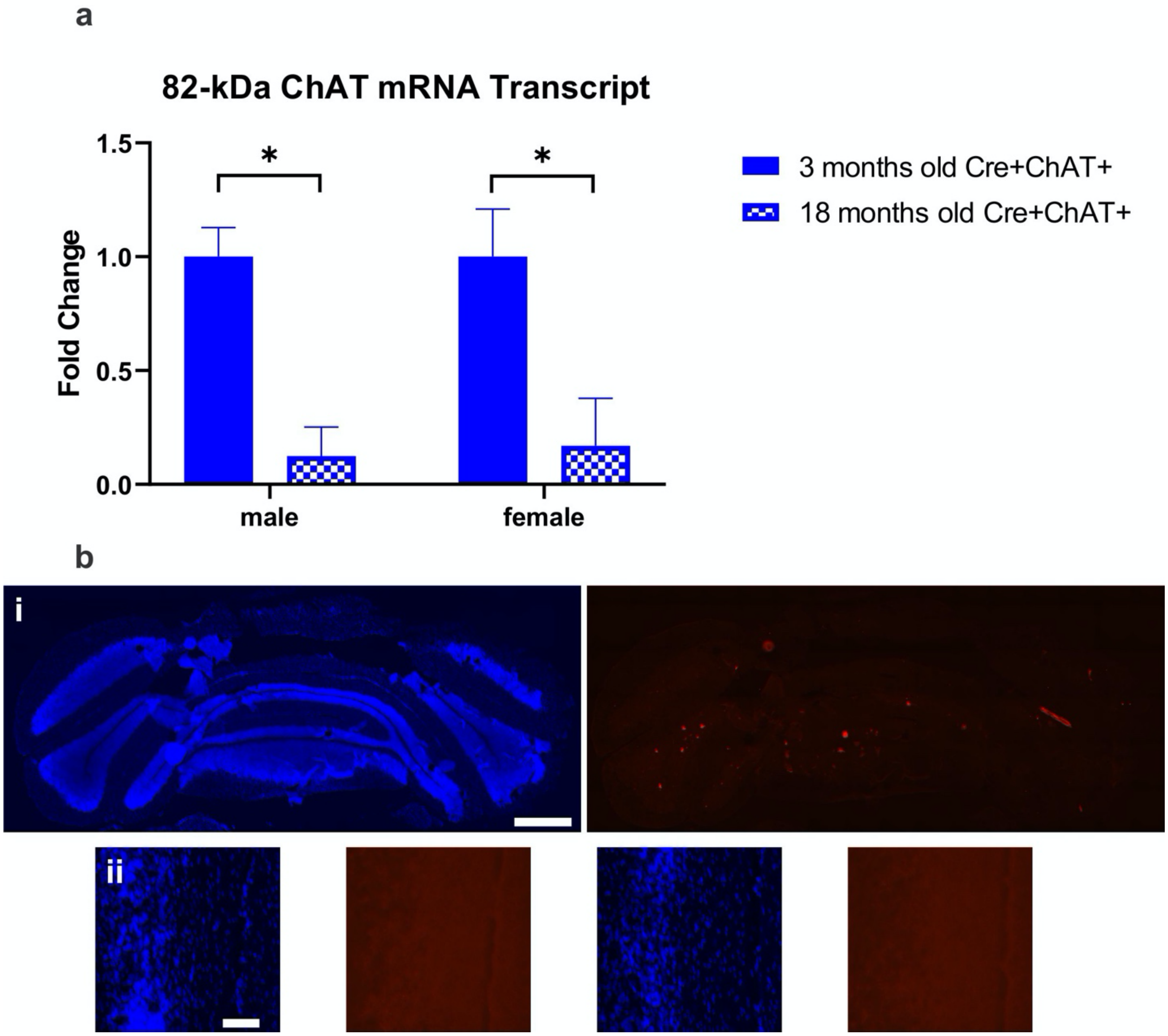

Supplementary Figure 2

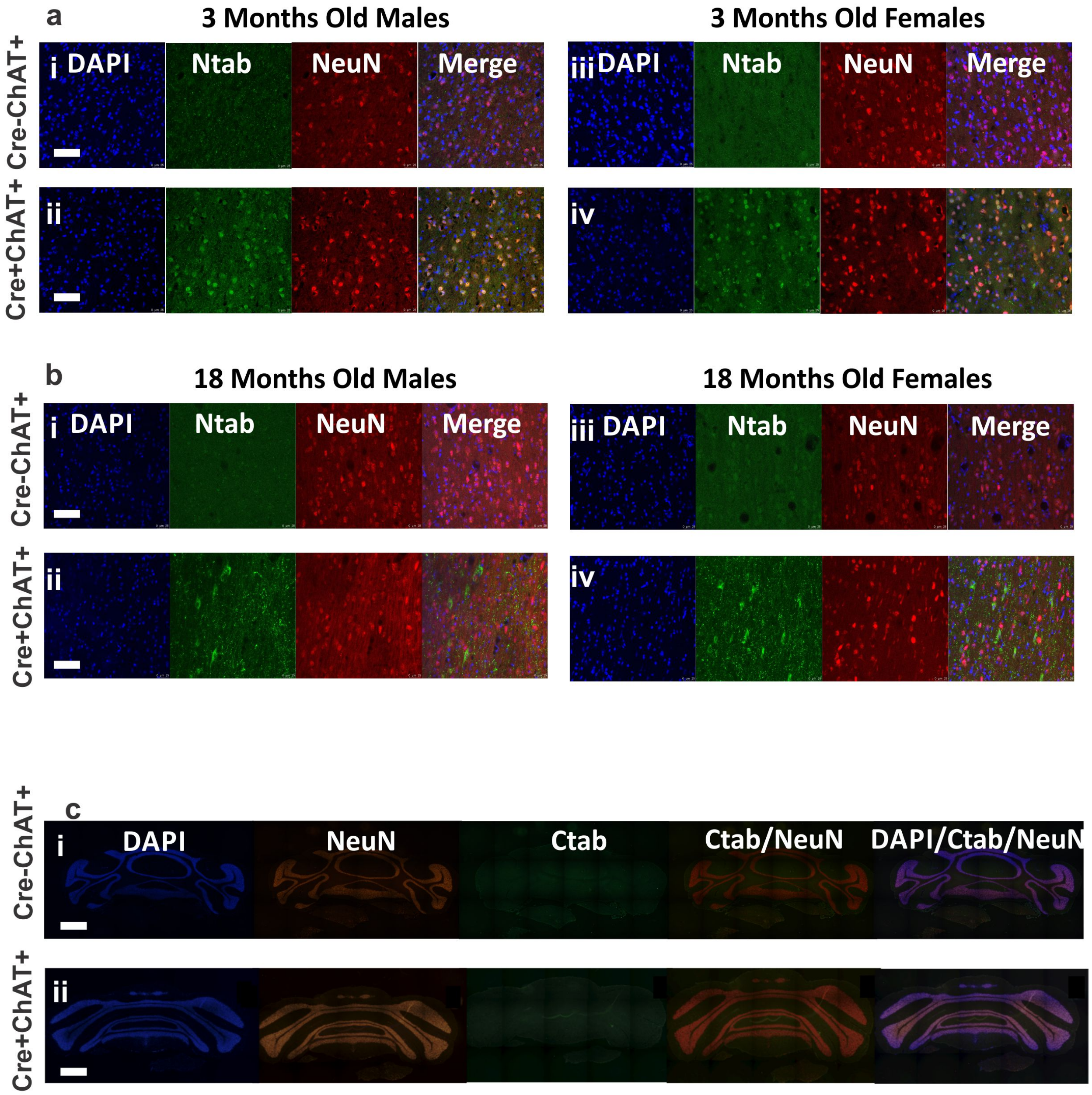

Supplementary Figure 3

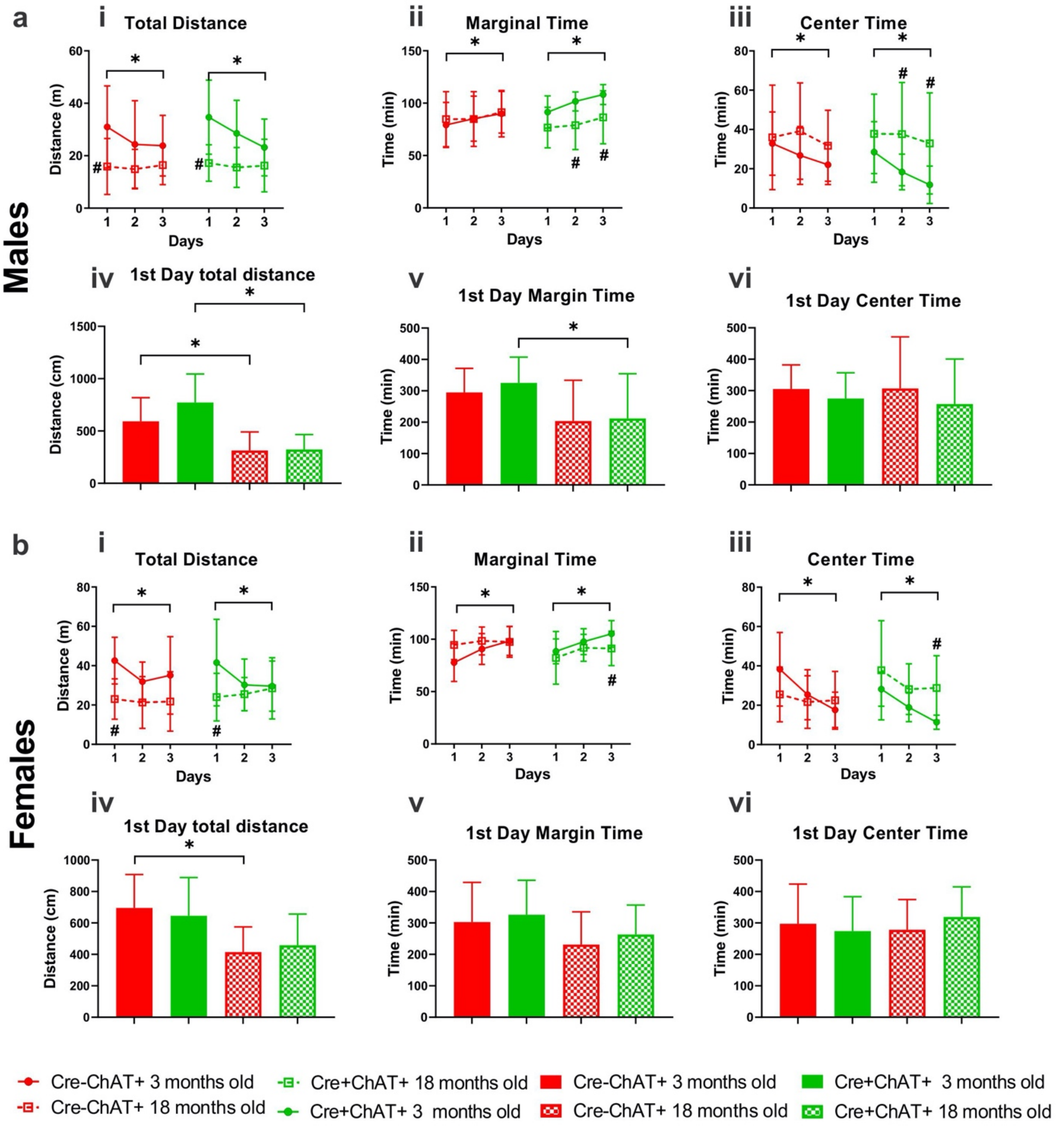

Supplementary Figure 4

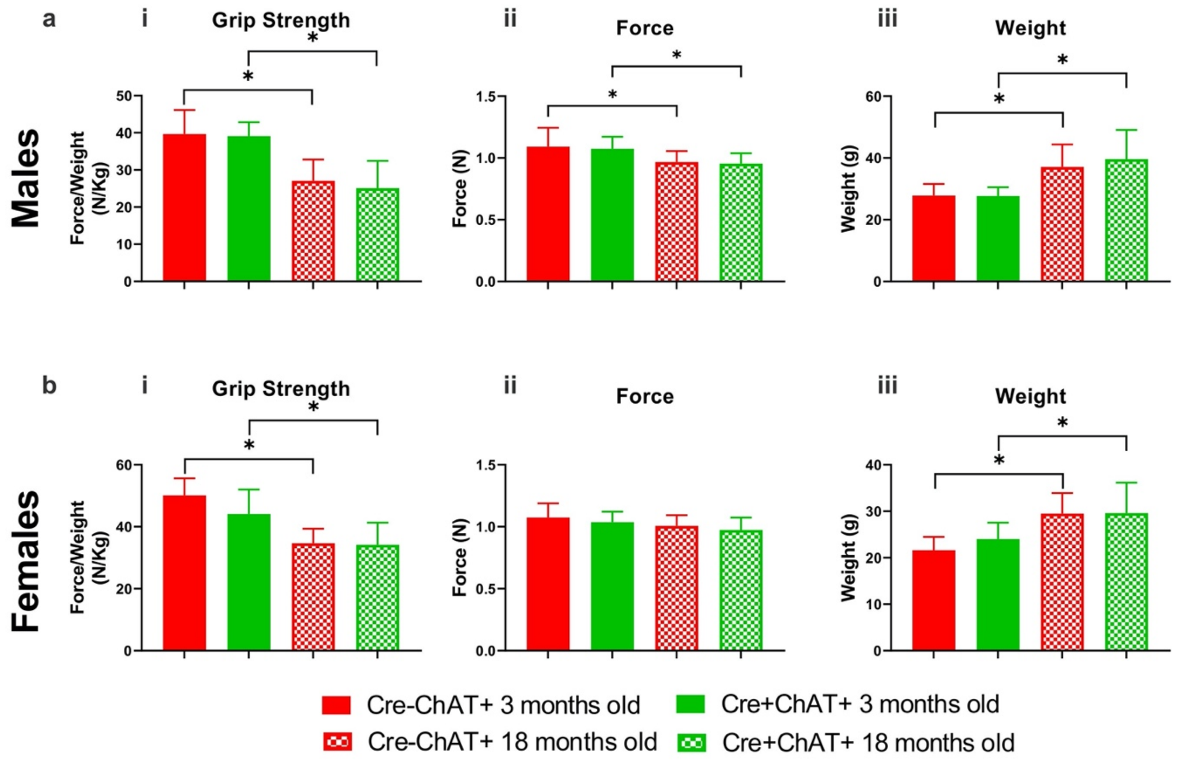

Supplementary Figure 5

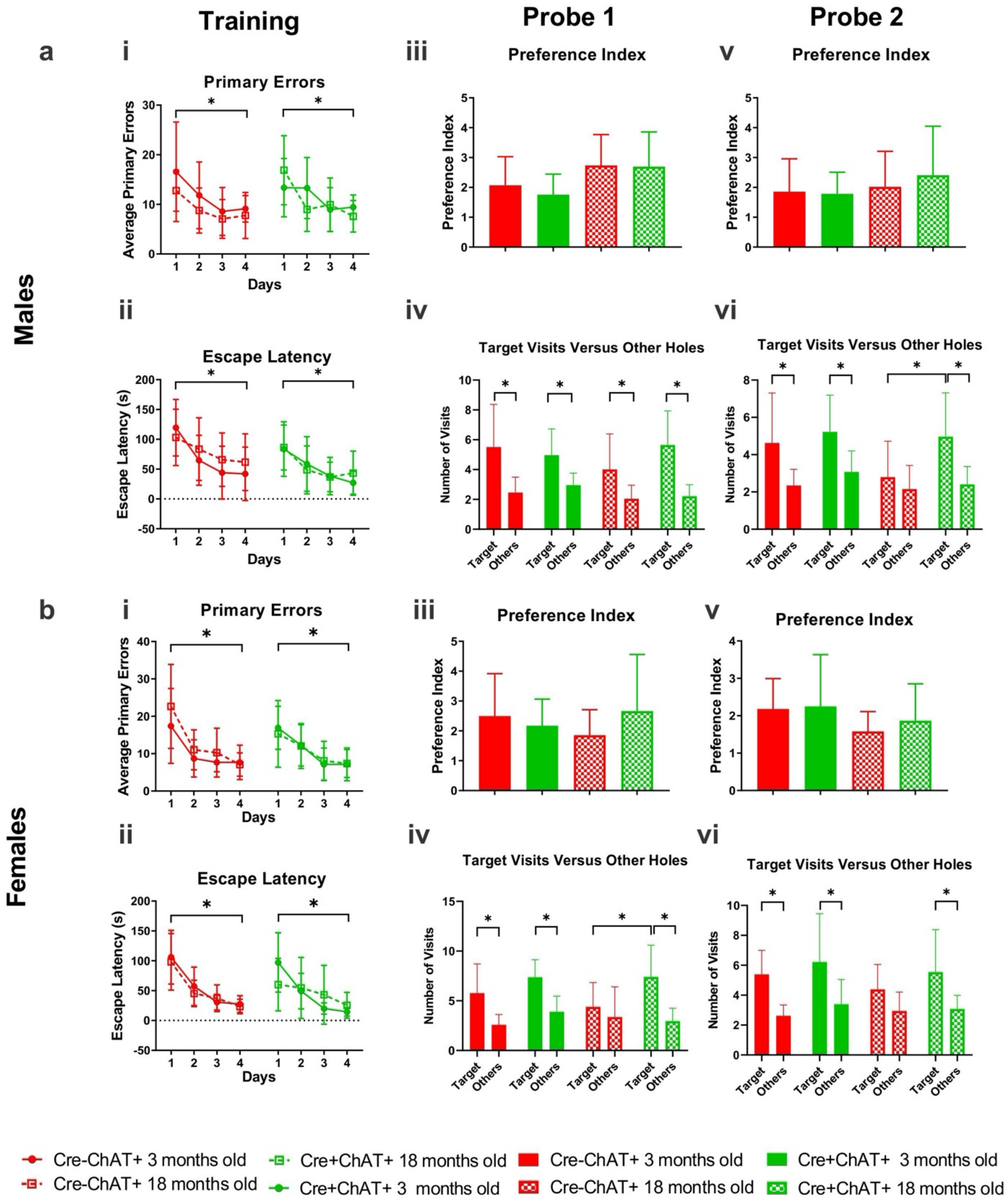

Supplementary Figure 6

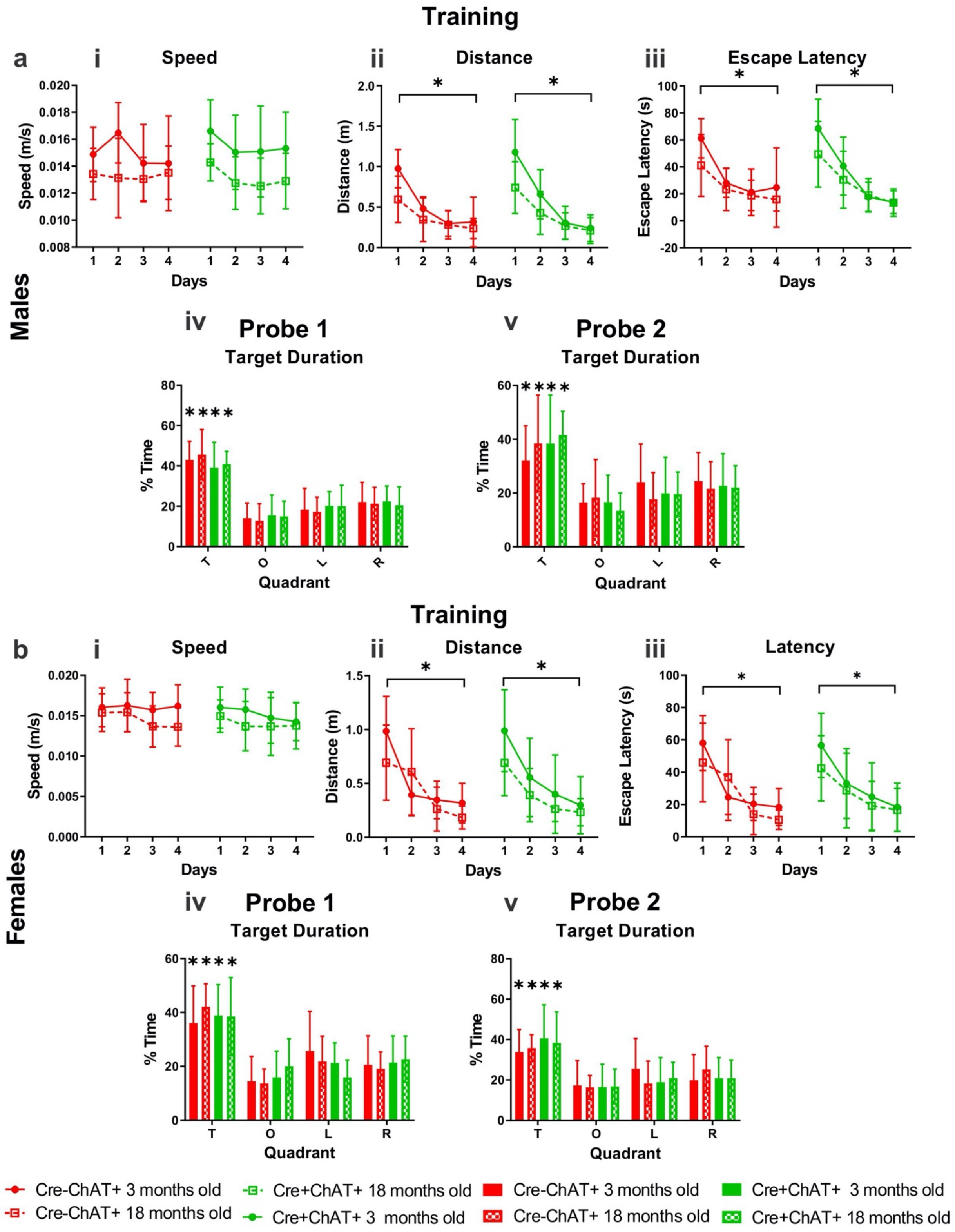

Supplementary Figure 7

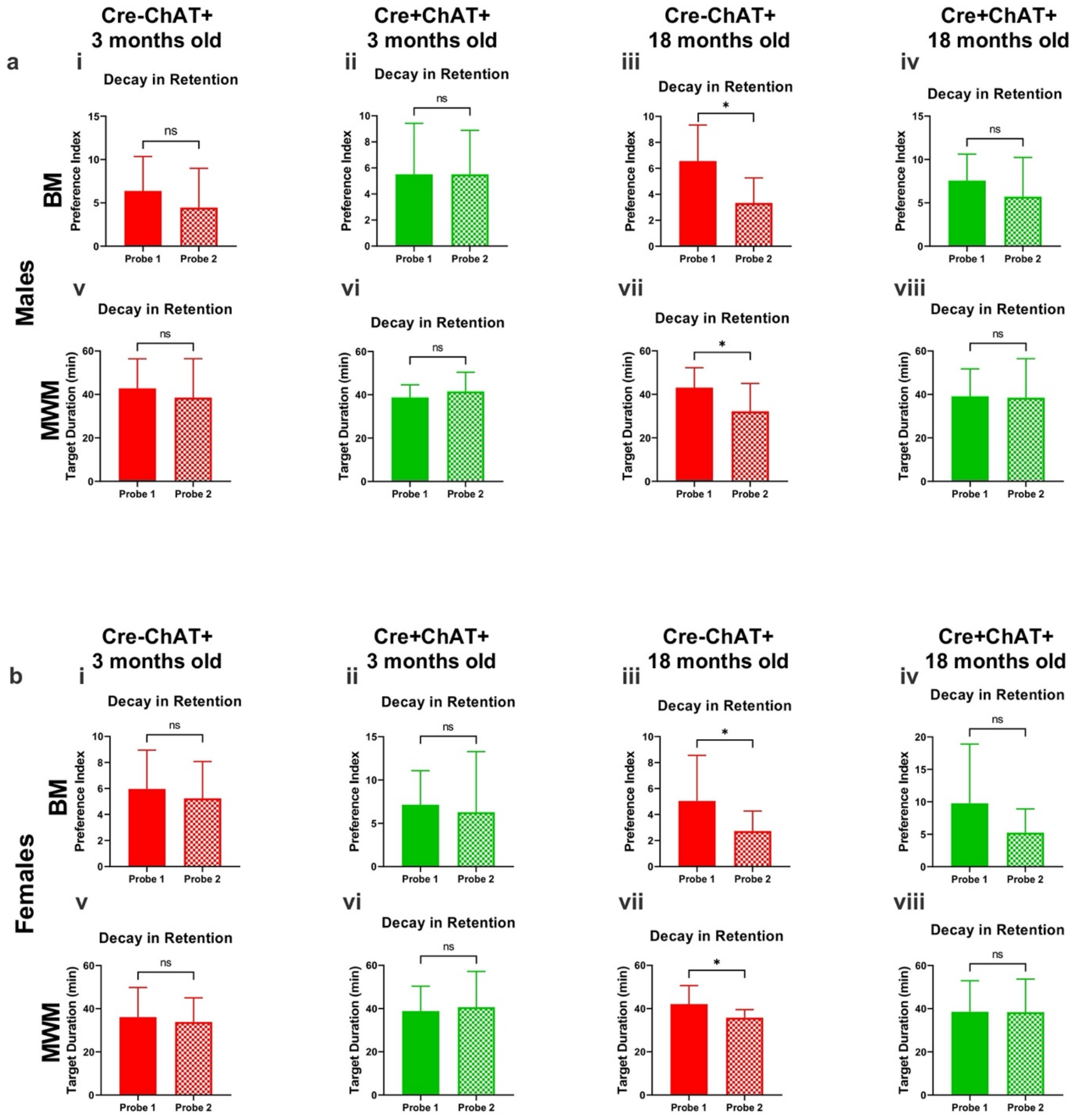

Supplementary Figure 8

Original Blots for Figure 6a: Males

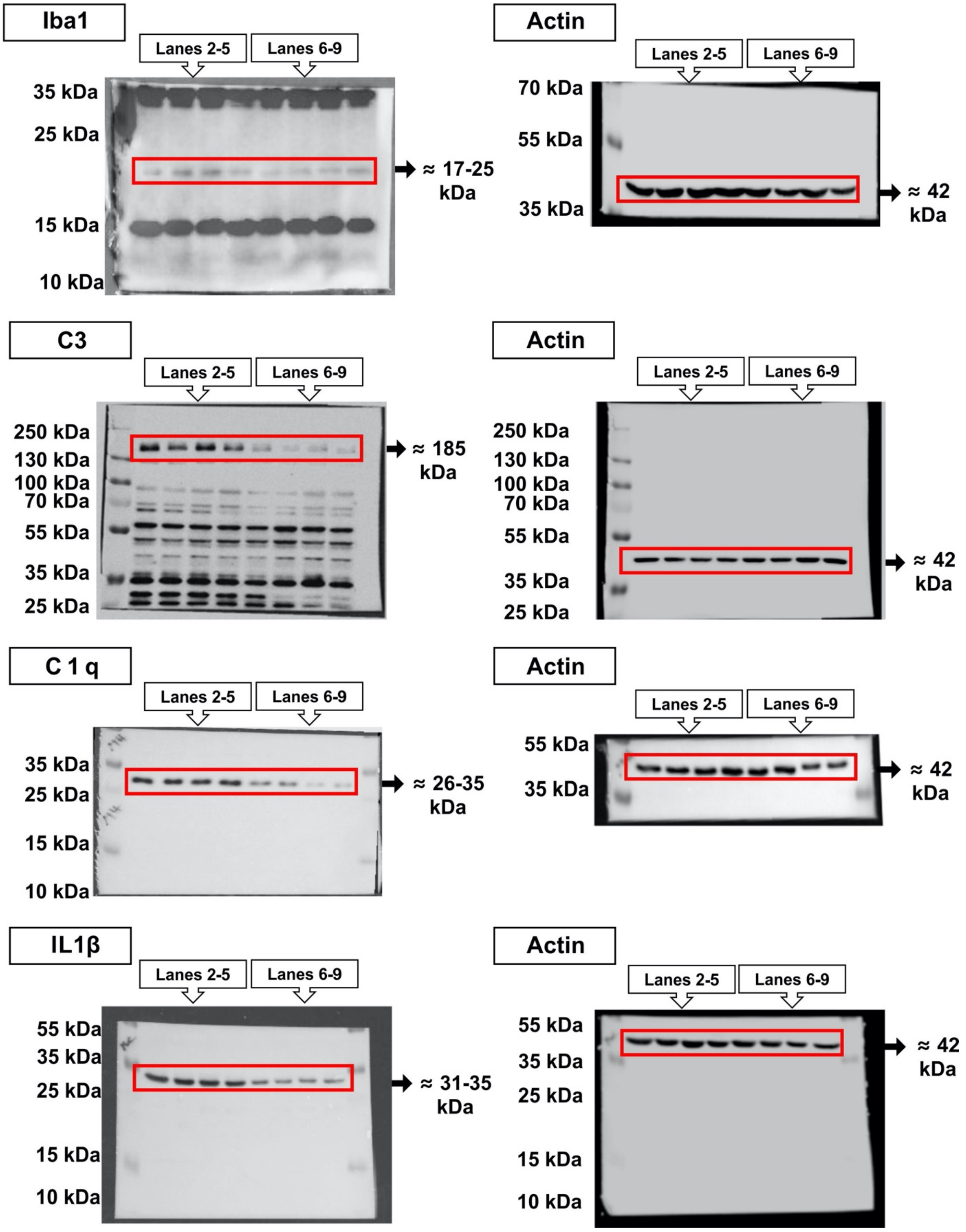

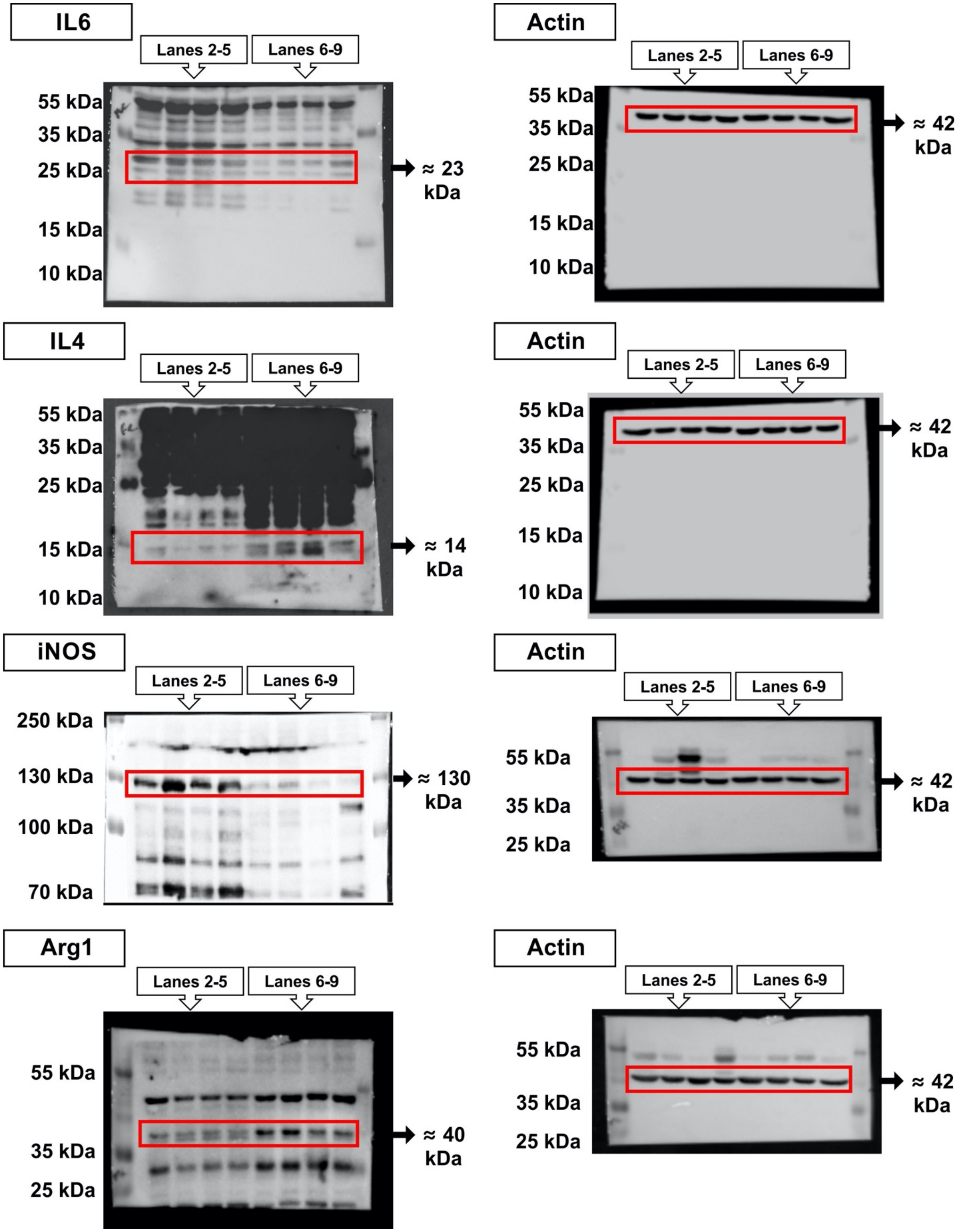
IL6
Lanes 2-5
Lanes 6-9
55 kDa
35 kDa
25 kDa
15 kDa
10 kDa
≈ 23 kDa
Actin
Lanes 2-5
Lanes 6-9
55 kDa
35 kDa
25 kDa
15 kDa
10 kDa
≈ 42 kDa
IL4
Lanes 2-5
Lanes 6-9
55 kDa
35 kDa
25 kDa
15 kDa
10 kDa
≈ 14 kDa
Actin
Lanes 2-5
Lanes 6-9
55 kDa
35 kDa
25 kDa
15 kDa
10 kDa
≈ 42 kDa
iNOS
Lanes 2-5
Lanes 6-9
250 kDa
130 kDa
100 kDa
70 kDa
≈ 130 kDa
Actin
Lanes 2-5
Lanes 6-9
55 kDa
35 kDa
25 kDa
≈ 42 kDa
Arg1
Lanes 2-5
Lanes 6-9
55 kDa
35 kDa
25 kDa
≈ 40 kDa
Actin
Lanes 2-5
Lanes 6-9
55 kDa
35 kDa
25 kDa
≈ 42 kDa

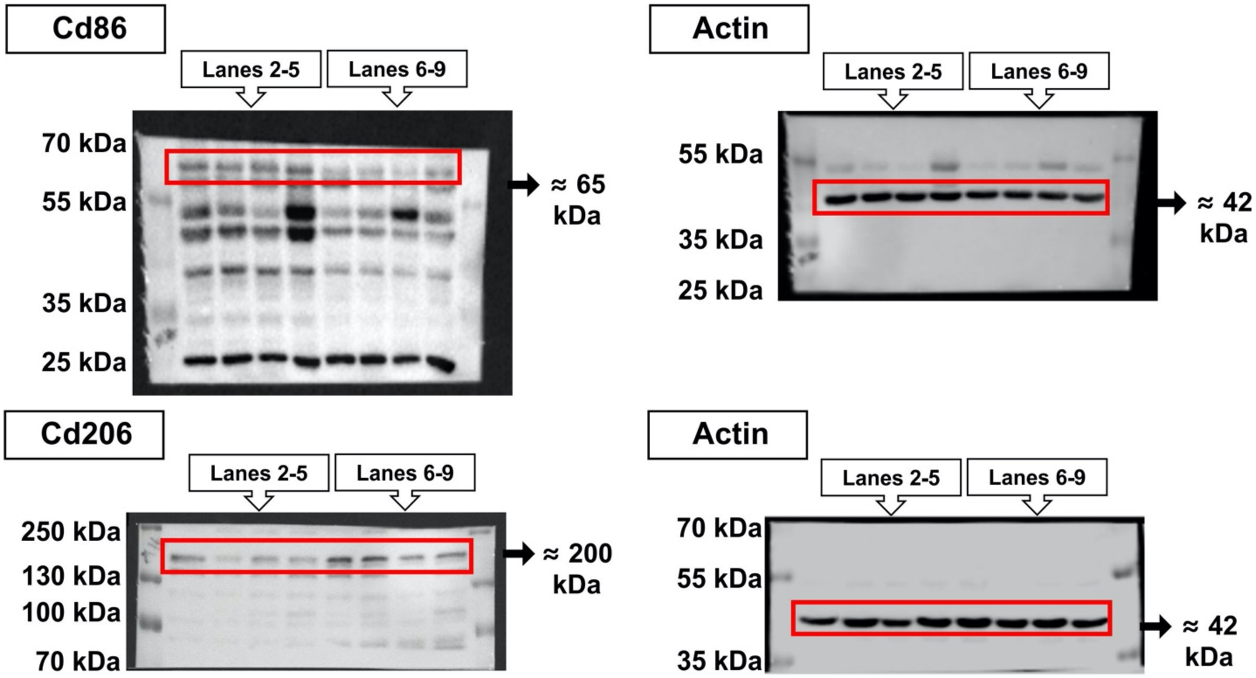
Cd86
Lanes 2-5
Lanes 6-9
70 kDa
55 kDa
35 kDa
25 kDa
≈ 65 kDa
Actin
Lanes 2-5
Lanes 6-9
55 kDa
35 kDa
25 kDa
≈ 42 kDa
Cd206
Lanes 2-5
Lanes 6-9
250 kDa
130 kDa
100 kDa
70 kDa
≈ 200 kDa
Actin
Lanes 2-5
Lanes 6-9
70 kDa
55 kDa
35 kDa
≈ 42 kDa

Supplementary Figure 9

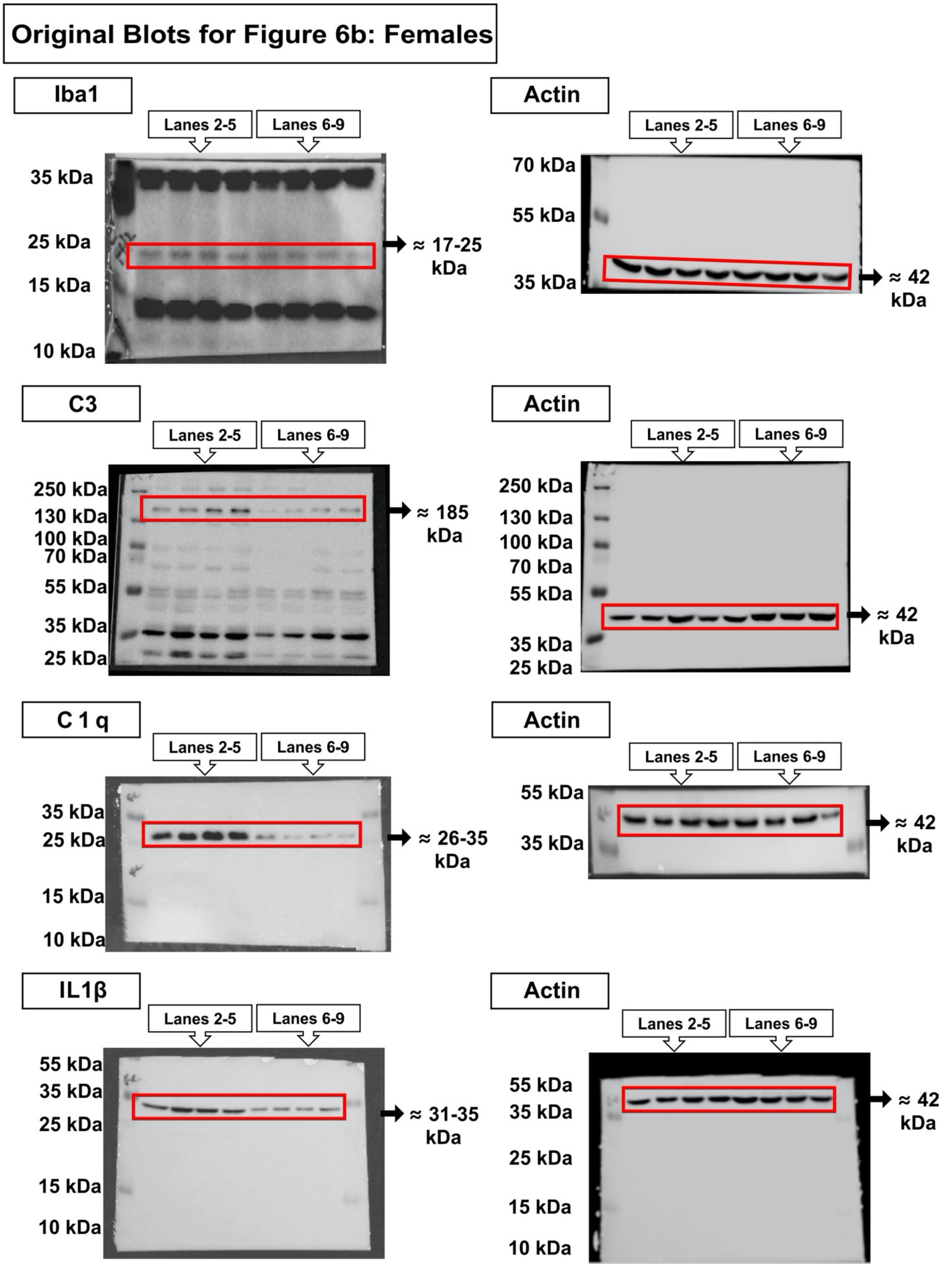

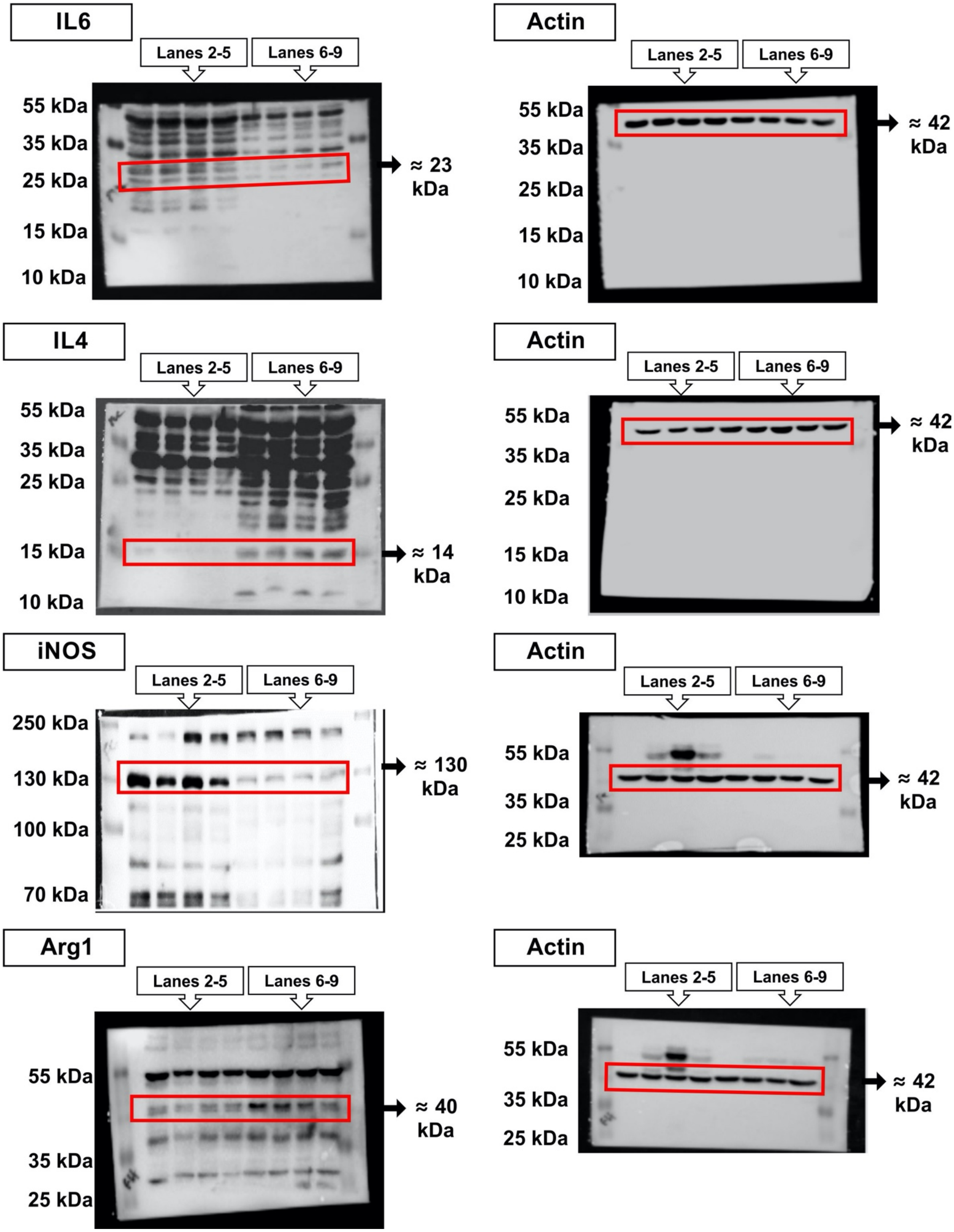
IL6
Lanes 2-5
Lanes 6-9
55 kDa
35 kDa
25 kDa
15 kDa
10 kDa
≈ 23 kDa
Actin
Lanes 2-5
Lanes 6-9
55 kDa
35 kDa
25 kDa
15 kDa
10 kDa
≈ 42 kDa
IL4
Lanes 2-5
Lanes 6-9
55 kDa
35 kDa
25 kDa
15 kDa
10 kDa
≈ 14 kDa
Actin
Lanes 2-5
Lanes 6-9
55 kDa
35 kDa
25 kDa
15 kDa
10 kDa
≈ 42 kDa
iNOS
Lanes 2-5
Lanes 6-9
250 kDa
130 kDa
100 kDa
70 kDa
≈ 130 kDa
Actin
Lanes 2-5
Lanes 6-9
55 kDa
35 kDa
25 kDa
≈ 42 kDa
Arg1
Lanes 2-5
Lanes 6-9
55 kDa
35 kDa
25 kDa
≈ 40 kDa
Actin
Lanes 2-5
Lanes 6-9
55 kDa
35 kDa
25 kDa
≈ 42 kDa

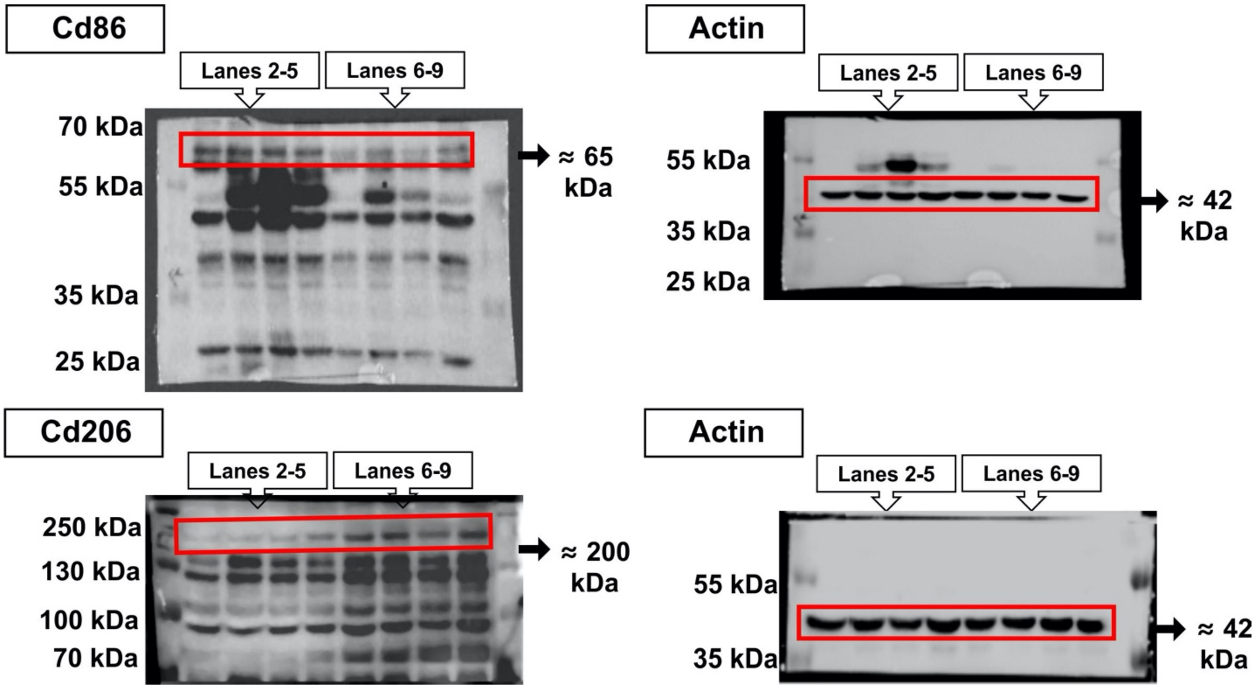
Cd86
Lanes 2-5
Lanes 6-9
70 kDa
55 kDa
35 kDa
25 kDa
≈ 65 kDa
Actin
Lanes 2-5
Lanes 6-9
55 kDa
35 kDa
25 kDa
≈ 42 kDa
Cd206
Lanes 2-5
Lanes 6-9
250 kDa
130 kDa
100 kDa
70 kDa
≈ 200 kDa
Actin
Lanes 2-5
Lanes 6-9
55 kDa
35 kDa
≈ 42 kDa
